# Single‐cell profiling reveals peripheral blood immune landscape remodelling in breast cancer lymph node metastasis

**DOI:** 10.1002/ctm2.70686

**Published:** 2026-06-09

**Authors:** Bo Chen, Kang Ma, Yunjie Wang, Liulu Zhang, Xinyue Feng, Cheng Long, Xuejing Tan, Kun Wang

**Affiliations:** ^1^ Department of Breast Cancer Cancer Center Guangdong Provincial People's Hospital Guangdong Academy of Medical Sciences Southern Medical University Guangzhou China; ^2^ School of Medicine South China University of Technology Guangzhou China; ^3^ Department of Pathology Yueyang Maternal Child Health‐Care Hospital Yueyang China

**Keywords:** breast cancer, lymphatic metastasis, peripheral blood, single‐cell analysis, tumour microenvironment

## Abstract

**Background:**

Axillary lymph node (LN) metastasis significantly impacts breast cancer (BC) prognosis. The role of the systemic immune environment in promoting metastasis remains unclear.

**Objectives:**

This study describes peripheral blood immune alterations associated with LN metastasis in BC patients.

**Methods:**

Peripheral blood mononuclear cells (PBMCs) from six BC patients with LN metastasis‐positive (Pos) and negative (Neg) underwent single‐cell RNA/T‐cell receptor sequencing (scRNA‐seq/scTCR‐seq). Findings were validated by in vitro functional experiments and single‐cell data from additional 14 BC tissues.

**Results:**

scRNA‐seq identified pro‐metastatic immune subpopulations enriched in Pos group: neutrophil_RSAD2 with expressing interferon‐stimulated genes (ISG) and neutrophil_MMP9 with expressing pro‐angiogenic factors, and immunosuppressive CD4Treg_FOXP3 and cytotoxic‐exhausted TandNK cells CD8Teff_GZMH and NKT_GNLY; and anti‐metastatic CD8Teff_CCL5 in Neg group. Pos group showed expanded large clonal T cells expressing cytotoxicity markers GZMH/GNLY. LGALS9‒HAVCR2 checkpoint interactions were elevated in Pos group through the neutrophil/mononuclear phagocyte (MP)‒MP/TandNK axis, which was associated with an immunosuppressive microenvironment and metastatic status. These pro‐ and anti‐metastatic immune signatures and LGALS9‒HAVCR2 axis were confirmed by in vitro experiments and consistently observed in BC tissues. Furthermore, functional co‐culture assays established a causal relationship: interferon‐stimulated neutrophils secreted LGALS9 and impaired their cytotoxic function on T cells. Conversely, neutralising LGALS9 or disrupting this axis restored T‐cell‐mediated tumour cell killing.

**Conclusion:**

LN metastasis is associated with a remodelled systemic immunity, enriches immunosuppressive neutrophils and TandNK cells and activates LGALS9‒HAVCR2 signalling. These findings reveal immune signatures associated with LN metastasis status and identify candidate biomarkers and therapeutic targets warranting further validation.

## Key points

- scRNA‐seq reveals pro‐metastatic neutrophil and exhausted T/NK subsets in BC patients with LN metastasis.
- Interferon‐stimulated neutrophils secrete LGALS9, drives T/NK cell exhaustion via HAVCR2.
- Neutralising LGALS9 restores T‐cell anti‐tumour activity, suggesting a novel therapeutic target.

## Graphical abstract

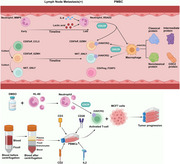

## 1 INTRODUCTION

Breast cancer (BC) is the neoplasm that exhibits the highest global burden of disease among women,^1^ while metastasis is the leading cause of death in BC patients.^2^, ^3^ Axillary lymph nodes (LNs) are the most frequent sites of metastasis for cancer cells,^4^ and the metastatic status is one of the significant prognostic factors for BC patients by affecting staging, treatment decisions and survival rates.^5^

Tumour microenvironment (TME) regulates carcinogenesis and progression by complicated interactions between cell surface proteins, secreted proteins and the corresponding ligand‒receptor (L‒R).^6^ Importantly, tumour‐draining lymph nodes (TDLNs) serve as a critical relay station connecting local metastasis to systemic immunity. Cancer cells in TDLNs actively remodel the local immune environment and can re‐enter the circulation, where they further modulate peripheral blood immune profiles.^7^, ^8^ TME consists of both stromal cells, which promote metastasis through angiogenesis, epithelial‒mesenchymal transition and invasion,^9^ and immune cells which can inhibit T‐cell activity to allow tumour cells elude immune surveillance.^10^ T cells are mainly recruited from the peripheral blood, rather than the original residence of TME.^11^ Peripheral blood T cells are driven by immunosuppression to switch functional states, such as resting Treg cell activation, effector T‐cell reduction and exhausted and suppressed T cells constant proliferation and cloning to suppress immune response.^12^ Notably, peripheral blood immune changes are not isolated events but reflect and interact with the primary TME's immunosuppressive landscape.^13^ The dynamic changes in circulating immune cells may indicate tumour immune state and metastatic potential non‐invasively.^14^ For instance, specific blood myeloid‐derived suppressor cell signatures linked with immunosuppressive TME and poor BC prognosis.^13^ Therefore, characterising peripheral blood immune signatures has translational potential and is convenient for dynamic disease monitoring, metastatic risk assessment and treatment response evaluation through routine blood sampling. Most studies have focused on local TME in primary or metastatic lesions,^6^, ^15^, ^16^ but the immune status of peripheral blood as a circulating reservoir for immune cells may determine tumour cell dissemination and colonisation after metastasis. The peripheral immune system and its role in immune escape in BC patients are still unclear. Recent single‐cell profiling of PBMCs in BC has begun to reveal metastasis‐associated systemic immune signatures. Previous study has identified that metastatic BC patients exhibit significant downregulation of adaptive immunity and impaired immunomodulatory communication pathways in circulating immune cells compared to non‐metastatic patients.^17^

Single‐cell RNA sequencing (scRNA‐seq) can examine the immune system and specific subpopulations in the dimension of individual cells.^18^, ^19^

We hypothesised that LN metastasis induces systemic remodelling of peripheral blood immune profiles, characterised by (i) enrichment of immunosuppressive neutrophil subsets expressing interferon‐stimulated genes (ISG) and pro‐angiogenic factors, (ii) accumulation of cytotoxic yet exhausted TandNK cells, and (iii) activation of the LGALS9‒HAVCR2 checkpoint axis that drives T‐cell exhaustion and promotes an immune‐permissive environment for metastasis. To test this hypothesis, we used scRNA‐seq to characterise systematic differences in immune cell composition, functional status, clonal structure, differentiation trajectory and intercellular communication network in the preoperative peripheral blood of LN metastasis‐positive (Pos) and negative (Neg) BC patients and how these differences shaped the systemic immune environment permitting or suppressing LN metastasis, which were validated by single‐cell data from BC tissues and in vitro functional experiments. We found that CD8Teff_CCL5 enrichment in Neg group might recruit effector cells to exert anti‐tumour effects, neutrophil_MMP9 and CD4Treg_FOXP3 enrichment in Pos group could promote tumour progression, and LGALS9‒HAVCR2 in Pos group was enriched among neutrophil_RSAD2, mononuclear phagocytes (MPs) and TandNK cells exhibiting dual cytotoxic‐exhausted characteristics such as CD8Teff_ GZMH and NKT_GNLY, and we further employed gain‐ and loss‐of‐function approaches in a co‐culture system to interrogate whether the LGALS9‒HAVCR2 axis drives T‐cell exhaustion and immunosuppression to promote tumour cells escape. Previous study has established that LGALS9 binds to HAVCR2 on T cells to induce immunosuppression, and that tumour‐associated neutrophils (TANs) can be polarised into pro‐tumourigenic phenotypes that support metastatic progression.^20^ By depicting the heterogeneity of immune cells with and without LN metastasis, this study provides a basis for prediction and treatment of metastatic BC patients in the future.

## 2 METHODS

### 2.1 Patient cohort and sample acquisition

#### 2.1.1 Patients

This study included 20 unpaired patients diagnosed with invasive luminal B subtype BC by puncture biopsy and pathological examination (three Pos and three Neg for scRNA/TCR‐seq of PBMC, seven Pos and seven Neg for scRNA for tumour tissues). Pre‐surgical imaging, intra‐surgical cytology and post‐surgical pathology confirmed LN metastasis, and all patients were free of acute or chronic disease that could affect the immune response. Specific clinical and pathological features could be found in Table S1. Every experimental procedure followed the Declaration of Helsinki and was authorised by the Guangdong Provincial People's Hospital Ethics Committee.

#### 2.1.2 Collection and separation of peripheral blood mononuclear cells

Preoperatively, 10 mL of the patient's peripheral blood was frozen (4°C) in an EDTA anticoagulant tube and sent to the laboratory. Three millilitres of venous blood and 3 mL of dulbecco's phosphate‐buffered saline (DPBS, Solarbio) were homogeneously combined in a 15 mL centrifuge tube. Prepared 3 mL of Ficoll (Solarbio) in another 15 mL centrifuge tube, carefully spread the diluted venous blood on it, and centrifuged at 500 *g* with the speed dropped to 0 for 30 min. After obvious delamination appeared, the leukoplakia layer's peripheral blood mononuclear cells (PBMC) were aspirated to a new centrifuge tube and resuspended with RBC Lysis Buffer (CWBIO). After centrifugation at 250 *g* for 10 min, the supernatant was discarded and the PBMC were resuspended in DPBS, washed again and formed as single‐cell suspensions. Then trypan blue was used to evaluate under the microscope to ensure that all newly isolated PBMC had cell viability levels of over 90%.

### 2.2 Single‐cell RNA sequencing and TCR sequencing library preparation

The methods of constructing scRNA‐seq and single‐cell TCR sequencing (scTCR‐seq) libraries have been mentioned in previous study.^19^ scTCR‐seq libraries were constructed using the Singleron GEXSCOPE system. TCR clonotypes were defined by the unique CDR3 amino acid sequences of TCRα and TCRβ chains. Clone size was categorised as single (clone size = 1), medium (1 < clone size ≤ 10) or large (clone size > 10), following established criteria.^21^ T‐cell diversity was estimated using Hill numbers (Chao1, D50, and Inverse Simpson index) as implemented in the iNEXT R package (version 3.0.0).

### 2.3 Bioinformatic analysis of single‐cell data

#### 2.3.1 Raw data reading and cell‐type identification

The original sequencing reads were processed using Celescope (Singleron Biotechnologies) with default parameters to generate gene expression count matrices. Ambient RNA contamination was removed using DecontX (version 1.6.1, within the celda package). Doublet detection and removal were performed using Scrublet (version 0.2.3) with default parameters. For batch effect correction across samples, Harmony (version 0.0.6) was applied. After these preprocessing steps, cells were filtered using the following quality control criteria: >500 genes per cell (nFeature_RNA), 2000–25 000 unique molecular identifier counts (nCount_RNA) and <10% mitochondrial gene content (percent.mt), as shown in Figure S1. Cell type annotation was performed using canonical markers from the SynEcoSysTM (Singleron Biotechnology) reference database. For clustering analysis, the Scanpy (version 1.9.3) toolkit was used with a resolution parameter of 1.0.

#### 2.3.2 Copy number variation analysis

Copy number variation (CNV) analysis was performed using inferCNV (version 1.8.1) to distinguish malignant epithelial cells from non‐malignant cells in BC tissue samples. A reference gene expression profile was constructed using non‐malignant cells (fibroblasts, endothelial cells and immune cells) from the same dataset. The analysis was run with default parameters (cutoff = .1, analysis_mode = ‘subclusters’, denoise = TRUE, HMM = FALSE). Epithelial cells exhibiting large‐scale chromosomal amplifications or deletions were classified as malignant.

#### 2.3.3 Differentially expressed gene analysis

For differential expression analysis, cells from Pos and Neg groups were compared using the Scanpy (version 1.9.3) rank_genes_groups function with default parameters. Differentially expressed genes (DEGs) were defined as those expressed in >10% of cells in either group, with |log_2_ fold change| > .25 and Benjamini‒Hochberg‐adjusted *p*‐value < .05.

#### 2.3.4 Pathway enrichment analysis

Gene Ontology (GO) enrichment analysis, which included gene sets for molecular function, biological process and cellular component, was used to showcase potential pathways of DEGs. Significantly enriched pathways were displayed using bar plot.

#### 2.3.5 Gene Set Enrichment Analysis

Gene Set Enrichment Analysis (GSEA) was based on MSigDB database, which was used to analyse the statistical significance of selected gene sets. The criteria for significantly enriched gene sets were |normalised enrichment score| > 1, *p*‐value < .05, and false discovery rate *q*‐value < .25.

#### 2.3.6 Ro/e analysis

The Ro/e ratio, defined as the ratio of observed cell count to expected cell count under a chi‐square distribution, was used to quantify cell type enrichment or depletion across groups, with Ro/e > 1 indicating enrichment.

#### 2.3.7 UCell gene sets scoring

UCell scoring, which calculates gene set scores using the Mann‒Whitney *U*‐statistic, was used to evaluate pathway or functional signatures in individual cells. The gene sets for ISG signature, pro‐angiogenic signature, cytotoxicity signature, exhaustion signature, proliferation signature and Treg‐specific signature were compiled from the MSigDB.

#### 2.3.8 Pseudo‐time trajectory analysis

Pseudo‐time trajectory analysis was performed using Monocle 2 (version 2.18.0). We selected Monocle 2 over newer tools such as Monocle 3 or RNA velocity because of its reversed graph embedding algorithm, which is well‐suited for identifying bifurcating differentiation trajectories in our dataset and trajectories are visualised by state or cell type to illustrate differentiation pathways.

#### 2.3.9 Cell‒cell communication analysis

Cell‒cell communication analysis was performed using CellPhoneDB (version 2.1.7), which provides a curated database of ligand‒receptor interactions and a statistical framework for inferring intercellular communication from scRNA‐seq data.^22^ For each ligand‒receptor pair, significance was assessed by a permutation test (1000 permutations) with a default expression threshold of 10% of cells expressing the gene in the cell type of interest. Interactions with *p* ≤  .05 were considered statistically significant. The interaction intensity was calculated based on the average expression of receptors and ligands in the cell population. We focused specifically on immune checkpoint interaction pairs to investigate immunosuppressive signalling networks.

#### 2.3.10 TCGA database analysis

Transcriptome data set came from TCGA‒BRCA cohort of TCGA database. After quality control and extraction, gene expression data of tumour patients with Luminal B subtype were selected and divided into LN metastasis group and non‐metastasis group. GraphPad software (version 9.2) was used to draw the gene expression difference plot, and *t* test was used to evaluate the statistical significance of the difference between the two groups.

### 2.4 In vitro and functional validation experiments

#### 2.4.1 Flow cytometric analysis

The PBMC suspension was incubated with Zombie Aqua‐BV510 (Biolegend) for 15 min at room temperature to exclude dead cells, 1 mL of Flow Cytometry Staining Buffer (Elabscience) was added and centrifuged at 300 *g* for 5 min, after discarding the supernatant, 100 µL of buffer was added and resuspended, CD3‐AF700, CD8‐BV421, CD45‐APC/Fire 750, CD56‐PE/Cy7 and CCR7‐FITC surface staining antibodies (Biolegend) were added sequentially and incubated at room temperature under light, washed and resuspended as before, after the membrane was broken and fixed according to the instructions of Intracellular Fixation/Permeabilisation Buffer (Elabscience), CCL5‐AF67 and GNLY‐PE intracellular staining antibodies (Biolegend) were added and incubated as before, washed and resuspended to prepare for use. Data were acquired by Cytexpert on Cytflex flow cytometric analyser (Beckman Coulter), and compensated and analysed by FlowJo. CD3‐AF700 and CD69‐BV650 surface staining antibodies (Biolegend) were used to identify activated T cells expanded by T‐cell activator in PBMC. Sequential gating was performed to select single, live and CD3^+^T cells, followed by identification of CD56+GNLY+ cells and CD8+CD45+CCR7‒CCL5+ cells.

#### 2.4.2 In vitro HL‐60 culture

The human myeloid leukemia cell line HL‐60 cells were identified by short tandem repeat analysis and certified by the supplier (Cas9X). Cells were cultured in IMDM (Cas9X) with 20% foetal bovine serum and 1% penicillin‒streptomycin in T25 culture flasks vertically in air with 5% CO_2_ at 37°C. HL‐60 cells were plated in six‐well plates with 1.25% dimethyl sulfoxide (DMSO, MP Bio) for 5 days to stimulate differentiation into neutrophil‐like cells.^23^, ^24^

#### 2.4.3 Western blot

After HL‐60 cells were cultured and differentiated into neutrophils‐like cells, different types and concentrations of type I interferon (IFN‐I, including IFN‐α, IFN‐β, Abclonal) were added to the DMSO‐free medium for stimulation, and the cells were collected 2 days later and lysed with RIPA lysate (Beyotime) containing protease inhibitor (PMSF, Proteintech). The specific experimental procedures of Western blot (WB) referred to the previous research.^25^ The primary antibodies used include MMP9, RSAD2, GAPDH, LGALS9 and HAVCR2 (Proteintech).

#### 2.4.4 Quantification of LGALS9 by enzyme‐linked immunosorbent assay

According to the instructions, the concentration of LGALS9 in neutrophil‐like cells stimulated by IFN‐I was detected by enzyme‐linked immunosorbent assay (human galectin 9 [GAL9] enzyme‐linked immunosorbent assay [ELISA] kit, Elabscience), and the results were displayed in pg/mL. Previous studies have suggested that BC patients with LN metastasis would increase inflammatory factors such as interleukin‐6 (IL‐6) and tumour growth factor‐beta (TGF‐β) or tend to be hypoxic.^26^, ^27^, ^28^ After the differentiation of HL‐60 cells induced by DMSO, TGF‐β, IL‐6 and L‐sodium lactate were added to six‐well plates to simulate the effects after LN metastasis.

#### 2.4.5 T‐cell activation and collection

The scheme of activating PBMC into T cells refers to the previous literature.^29^ PBMC cells were inoculated in RPMI Medium 1640 basic, Gibco) at a cell counting chamber slide (Countess) of 1 × 10^6^ cells/mL, and 20 ng/mL of the cytokine IL‐2 (Abclonal) and 25 µL/mL of immune Cult Human CD3/CD28/CD2 T‐cell activator (STEM Cell Technologies), then the cells were incubated in an incubator with 37°C and 5% CO_2_, and then the cell concentration was readjusted to 1 × 10^6^ with fresh culture medium every 2‒3 days, and cytokines and T‐cell activator should be added to the culture medium again.

#### 2.4.6 Transfection plasmid and siRNA

HL‐60 or activated T cells were washed and centrifuged with phosphate‐buffered saline (PBS), and then inoculated into a six‐well plate with Opti‐Mem (Gibco) at a density of 1 × 10^6^ per 1.5 mL. An amount of 125 µL Opti‐MEM and 5 µL Lipofectamine 3000 (Invitrogen) were added to tube 1, and 125 µL Opti‐MEM and 2.5 µg plasmid or 5 µL siRNA (Suzhou Hongxun Biotechnology Co., Ltd.) were added to tube 2. After mixing tube 1 and tube 2 at room temperature for 15 min, they were added to a six‐well plate, RNA was extracted for 36 h, and protein was extracted for 48 h.

#### 2.4.7 Co‐culture system

We used Transwell chamber (Corning, aperture  .4 µm) for co‐culture. HL‐60 cells were collected 48 h after transfection and inoculated into the upper chamber of Transwell chamber at a density of 1 × 10^6^, and the medium was serum‐free IMDM medium. The activated T cells were inoculated into the lower chamber at a density of 1 × 10^6^, and the culture medium was RPMI‐1640 medium containing cytokines and T‐cell activators. MCF‐7 cells purchased from ATCC were inoculated into the lower chamber at a density of 5 × 10^5^. The function and phenotype of the co‐culture system were detected after 48 h.

#### 2.4.8 qRT‐PCR

Total RNA was extracted using the RNA extraction kit (GOONIE) according to the manufacturer's instructions. The procedure of quantitative reverse transcription polymerase chain reaction (qRT‐PCR) was carried out according to the previous report.^30^ Primer sequences used were human LGALS9—forward: 5′‐CGTGTGGACACCATCTCCG‐3′, reverse: 5′‐CAGCCCTCCCAGAATGGTG‐3′; human HAVCR2—forward: 5′‐ TGTGATTGTAGATTTGGTAGTG‐3′, reverse: 5′‐CCTCTATACAACACCATTATATC‐3′; human ACTB—forward: 5′‐GGACTTCGAGCAAGAGATGG‐3′, reverse: 5′‐AGCACTGTGTTGGCGTACAG‐3′.

#### 2.4.9 Wound‐healing assay

Together with co‐culture, a sterile 200 µL pipette tip was used to create a uniform, straight scratch across the centre of each well. The wells were gently washed with PBS to remove detached cells and then replenished with fresh serum‐free medium to minimise proliferation effects. Images of the wound were captured at the same location for each well at 0 and 24 h post‐scratch using an inverted phase‐contrast microscope (Olympus). The wound area was measured using ImageJ software (National Institutes of Health), and the percentage of wound closure was calculated as follows: migration rate (%) = [(Area_t_0 h ‒ Area_t_24 h)/Area_t_0 h] × 100%.

#### 2.4.10 Colony formation assay

Together with co‐culture, MCF‐7 cells were inoculated into the lower chamber of a six‐well plate at a density of 1.5 × 10^3^. After 7–10 days of cell culture, the cells were washed with PBS, fixed with 4% paraformaldehyde for 15 min, and stained with  .5% crystal violet (Sigma‒Aldrich) for 30 min at room temperature. After full air drying, the colonies containing more than 50 cells were counted with ImageJ. The plating efficiency = (number of colonies formed/number of cells seeded) × 100%.

#### 2.4.11 Statistical analysis

Statistical analysis was performed using R 4.4.1 software. The Wilcoxon rank sum test and the paired or unpaired Student's *t*‐test were used to examine continuous variables, while the Fisher exact test or the chi‐square test was used to examine categorical variables. *p *< .05 indicated the difference was statistically significant. All single‐cell sequencing data were newly generated for this study and were not from public repositories.

## 3 RESULTS

### 3.1 Cellular profiling of patients' PBMCs

**FIGURE 1:**
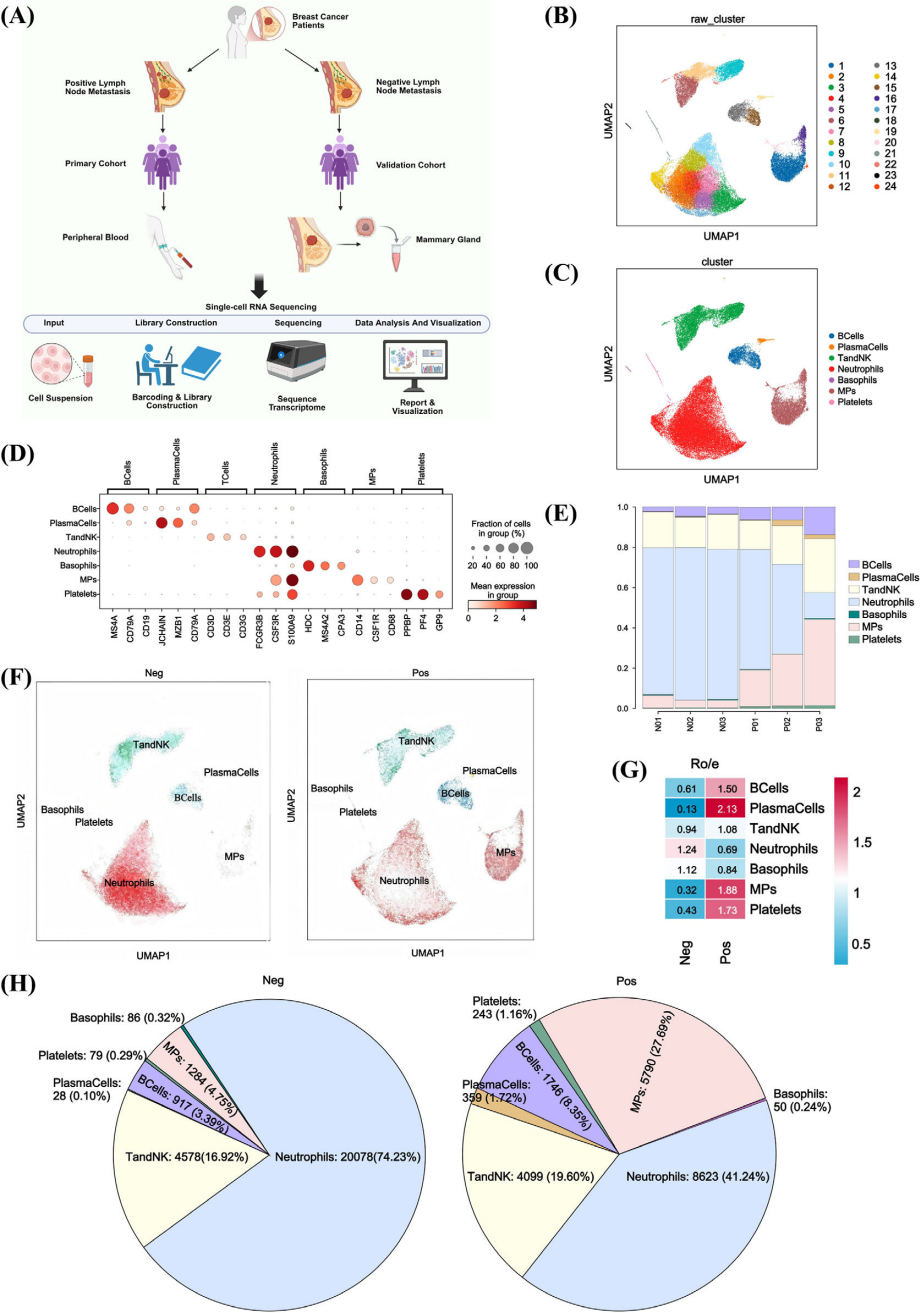
Single‐cell sequencing analysis of peripheral blood mononuclear cells (PBMCs). (A) Schematic flowchart of single‐cell sequencing and study design. (B) Clustering of all single cells using Uniform Manifold Approximation and Projection (UMAP) plot. (C) Visualisation of all annotated cell clusters. (D) Unbiased gene expression profiles of annotated cell clusters shown by dot plot. The dot plot showing the top three genes, identified by log‐fold change (FC), for each cluster of cells; the size of the dots indicating the percentage of cells in a cluster expressing the corresponding gene, and the colour of the dots reflecting the level of expression of the corresponding gene. (E) Proportional distribution of cell clusters between samples. (F) Distribution of PBMCs in breast cancer (BC) patients with lymph node (LN) metastasis‐positive (Pos) and negative (Neg). (G) Ro/e heatmap showing ratio of observed to expected cell counts. (H) Proportional distribution of cell clusters between groups through pie charts.

We first collected PBMC from unpaired six Pos and Neg BC patients before operation for scRNA‐seq and scTCR‐seq, and subsequently validated the results obtained by in vitro experiments and single‐cell data from additional BC tissues (Figure 1A). According to the standard procedure and quality control (Figure S1), the single‐cell data of PBMC were processed and yielded the transcriptome profile of 47 960 cells (Figure 1B). The conventional cellular markers and genes with upregulated expression identified 2663 B cells, 387 plasma cells, 8677 TandNK cells, 28 701 neutrophils, 136 basophils, 7074 MPs and 322 platelets (Figure 1C,D). All types of cells in the same group of LN metastasis accounted for approximately the same (Figure 1E). Observing the enrichment of TandNK cells, B cells and MPs and the decrease of neutrophils in Pos group and considering the potential functional importance of these immune cells, we next sought to characterise their heterogeneity at a higher resolution (Figure 1F‒H).

### 3.2 Neutrophils exhibiting higher ISG signature in Pos group

**FIGURE 2:**
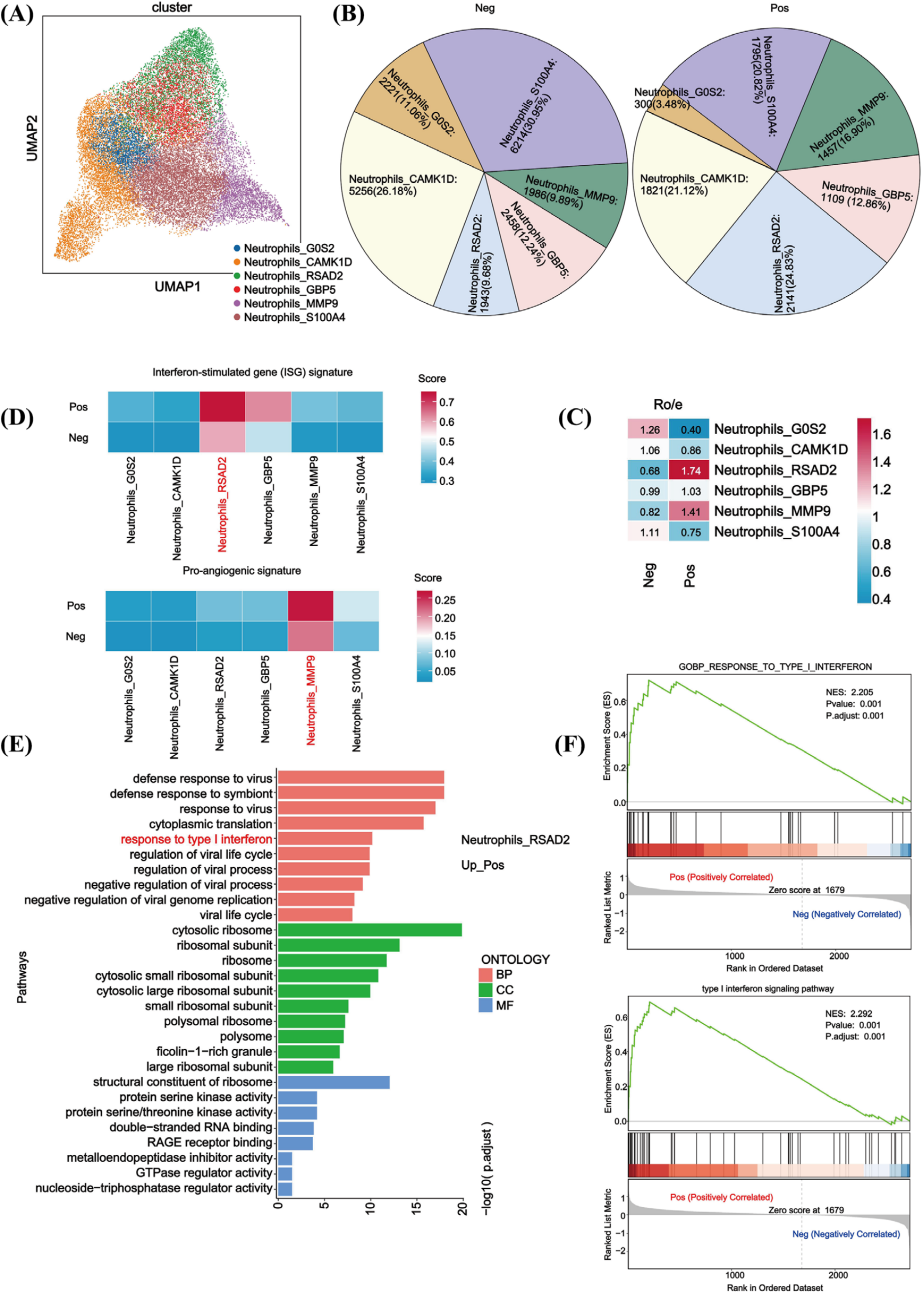
Neutrophils exhibiting higher interferon stimulation profile. (A) Visualisation of all annotated neutrophil clusters. (B) Proportional distribution of neutrophil clusters between groups through pie charts. (C) Ro/e heatmap showing ratio of observed to expected neutrophil counts. (D) Heatmap showing interferon‐stimulated gene (ISG) and pro‐angiogenic signatures characterising neutrophil clusters. (E) Gene Ontology (GO) enrichment analysis revealing upregulated pathways of neutrophils_RSAD2 in positive (Pos) group. (F) Gene Set Enrichment Analysis (GSEA) enrichment plots showing the enriched gene ontology biological process (GOBP) terms of response to type I interferon and the selected biological process of type I interferon signalling pathway significantly in neutrophils_RSAD2 within Pos group compared to negative (Neg) group.

Neutrophils were subdivided into six clusters and annotated as Neutrophil_G0S2, Neutrophil_CAMK1D, Neutrophil_RSAD2, Neutrophil_GBP5, Neutrophil_MMP9 and Neutrophil_S100A4 based on the typical markers (Figures 2A and S2A). While each cell type was similar within the group, neutrophil_RSAD2 and neutrophil_MMP9 were much more abundant in Pos group (Figures 2B,C and S2B,C). Similarly, the expression of RSAD2 and MMP9 genes was mainly concentrated in Pos group in neutrophils of large class annotations (Figure S2D). UCell scoring of selected specific gene sets for the annotated neutrophil subpopulations showed that neutrophil_RSAD2 scored higher than the other subclusters for ISG signature, which was significantly enriched in Pos group; neutrophils_MMP9 was the same as above for pro‐angiogenic signature (Figures 2D and S2E). The DEGs and GO enrichment analysis of neutrophil _RSAD2 and neutrophil _MMP9, mainly focusing on genes upregulated in Pos group, revealed that Neutrophil_RSAD2 in Pos group was enriched in response to IFN‐I, consistent with high ISG signature; and neutrophil_MMP9 was enriched in RAGE receptor binding, secretory granule lumen and cytoplasmic vesicle lumen, suggesting extracellular matrix (ECM) remodelling and secretion of pro‐angiogenic factor^31^ (Figures 2E and S2F,G). GSEA analysis of the enriched gene sets further illustrated that neutrophil _RSAD2 could react to IFN‐I and enrich IFN‐I pathway in Pos group compared with Neg group (Figure 2F). To understand the developmental relationship and dynamic gene expression changes among these functionally distinct subsets, we performed pseudo‐time trajectory analysis. All neutrophils differentiated into five states and two fate‐determining directions (Figure S2H). Both MMP9 and RSAD2 genes were highly variable genes, and MMP9 was mainly located in the early stage of differentiation corresponding to neutrophil _MMP9 at the beginning of differentiation, while RSAD2 was mainly located in the late stage of differentiation corresponding to neutrophil _RSAD2 at the ending of differentiation, and both cells were more concentrated in Pos group, which might suggest their potential association with LN metastasis (Figure S2I‒K). Besides, we performed a time‐course differentiation experiment and the results showed that MMP9 increased rapidly, peaking at day 3 and gradually declining thereafter, and in contrast, RSAD2 remained minimal until day 3, then rose sharply and reached its highest level at day 5 (Figure S2L). This spatiotemporal pattern suggests that neutrophil_MMP9 may initiate pro‐angiogenic and matrix remodelling functions early in differentiation, while neutrophil_RSAD2, emerging later, contributes to sustained interferon response and likely serves as the cellular source of LGALS9 that activates the LGALS9‒HAVCR2 axis in the metastatic microenvironment.

### 3.3 Characteristic TandNK cells in peripheral blood of Pos and Neg groups

**FIGURE 3:**
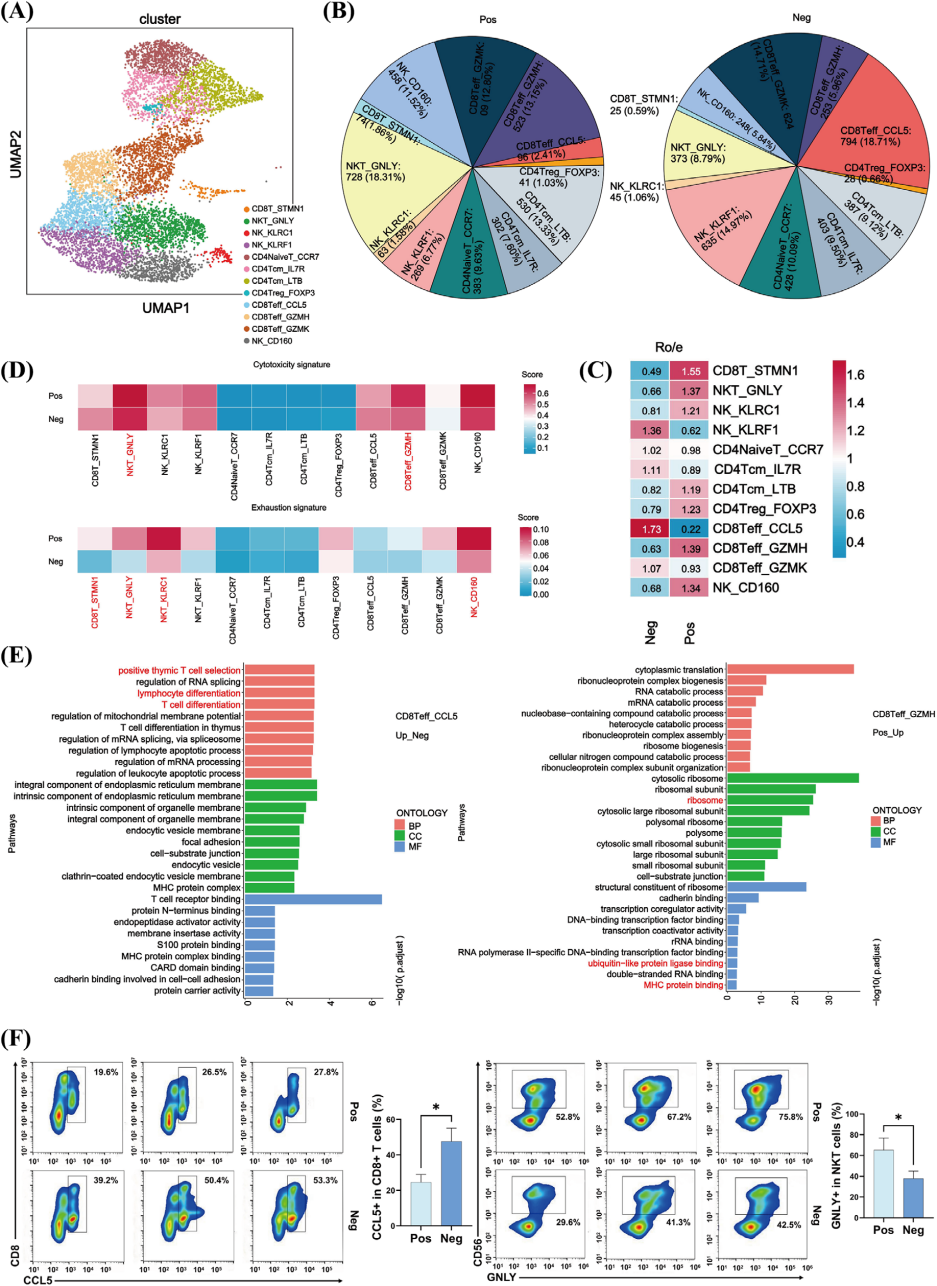
T cells having different immune responses. (A) Visualisation of all annotated T‐cell clusters. (B) Proportional distribution of T‐cell clusters between groups through pie charts. (C) Ro/e heatmap showing ratio of observed to expected T‐cell counts. (D) Heatmap showing cytotoxicity and exhaustion signatures characterising T‐cell clusters. (E) Gene Ontology (GO) enrichment analysis revealing upregulated pathways of CD8Teff_CCL5 and NKT_GNLY in negative (Neg) and positive (Pos) groups. (F) Representative flow cytometry images and statistics of CD3+CD8+CD45+CCR7‒CCL5+ cells and CD3+CD56+GNLY+ cells. T test was conducted.

TandNK cells were subdivided into CD8_STMN1, NKT_GNLY, NK_KLRC1, NK_KLRF1, CD4NaiveT_CCR7, CD4Tcm_IL7R, CD4Tcm_LTB, CD4Treg_FOXP3, CD8Teff_CCL5, CD8Teff_GZMH, CD8Teff_GZMK and NK_CD160 (Figures 3A and S3A). The proportions of each cell type were roughly the same within groups, but CD4Treg_FOXP3, CD8Teff_GZMH and NKT_GNLY were significantly higher and enriched in Pos group, whereas CD8Teff_CCL5 was mainly enriched in Neg group (Figures 3B,C and S3B‒D). FOXP3, GZMH and GNLY genes were prevalent and enriched in Pos group, while CCL5 genes in Neg group in large class annotations (Figure S3E). UCell scoring revealed that both CD8Teff_GZMH and NKT_GNLY exhibited high proliferation, cytotoxicity and exhaustion signatures, and CD4Treg_FOXP3 exhibited high Treg specific signature, which all were significantly enriched in Pos group (Figures 3D and S3F,G). GO enrichment analysis of DEGs upregulated by CD4Treg_FOXP3, CD8Teff_GZMH and NKT_GNLY in Pos group and CD8Teff_CCL5 in Neg group showed that CD4Treg_FOXP3 was enriched in focal adhesion and homeostasis of number of cells, which might indirectly promote tumour immune escape and metastasis by maintaining the functional stability and migratory ability of Treg cells^32^; CD8Teff_GZMH and NKT_GNLY were enriched in ribosome‐associated pathway, MHC protein binding and ubiquitin‐like protein ligase binding, possibly indicating that although toxic proteins were continuously secreted, losing lethality due to sustained antigenic exposure might promote the solidification of exhausted phenotypes and act as pro‐oncogenic factors in the metastatic microenvironment^33^, ^34^; CD8Teff_CCL5 might optimise immune surveillance through the enriched pathways of positive thymic T‐cell selection, T‐cell differentiation and lymphocyte differentiation^35^ (Figures 3E and S3H,I). Flow cytometry analysis of peripheral blood from six BC patients showed a notable rise in CD3+CD56+GNLY+ cells in Pos group and CD3+CD8+CD45+CCR7‒CCL5+ cells in Neg group, in line with the conclusions obtained in the single‐cell data (Figure 3F). T‐cell differentiation had five states and two fate‐determining directions, and GNLY and CCL5 genes were highly variable genes, and both were located in the early stage of differentiation and same fate direction, while NKT_GNLY was more enriched in Pos group and CD8Teff_CCL5 in Neg group, which further confirmed the above conclusions and suggested that Pos and Neg groups possessed own characteristic TandNK cells as markers (Figure S3J‒M). The early enrichment of CCL5 in Neg group and GNLY in Pos group aligns with their respective roles in immune recruitment and cytotoxicity. Notably, GNLY+ cells in Pos group, which are prone to exhaustion, represent the terminal effectors influenced by the LGALS9‒HAVCR2 axis, linking early differentiation commitment to later dysfunctional states.

### 3.4 Amplification of clonal T cells in peripheral blood of Pos and Neg groups

**FIGURE 4:**
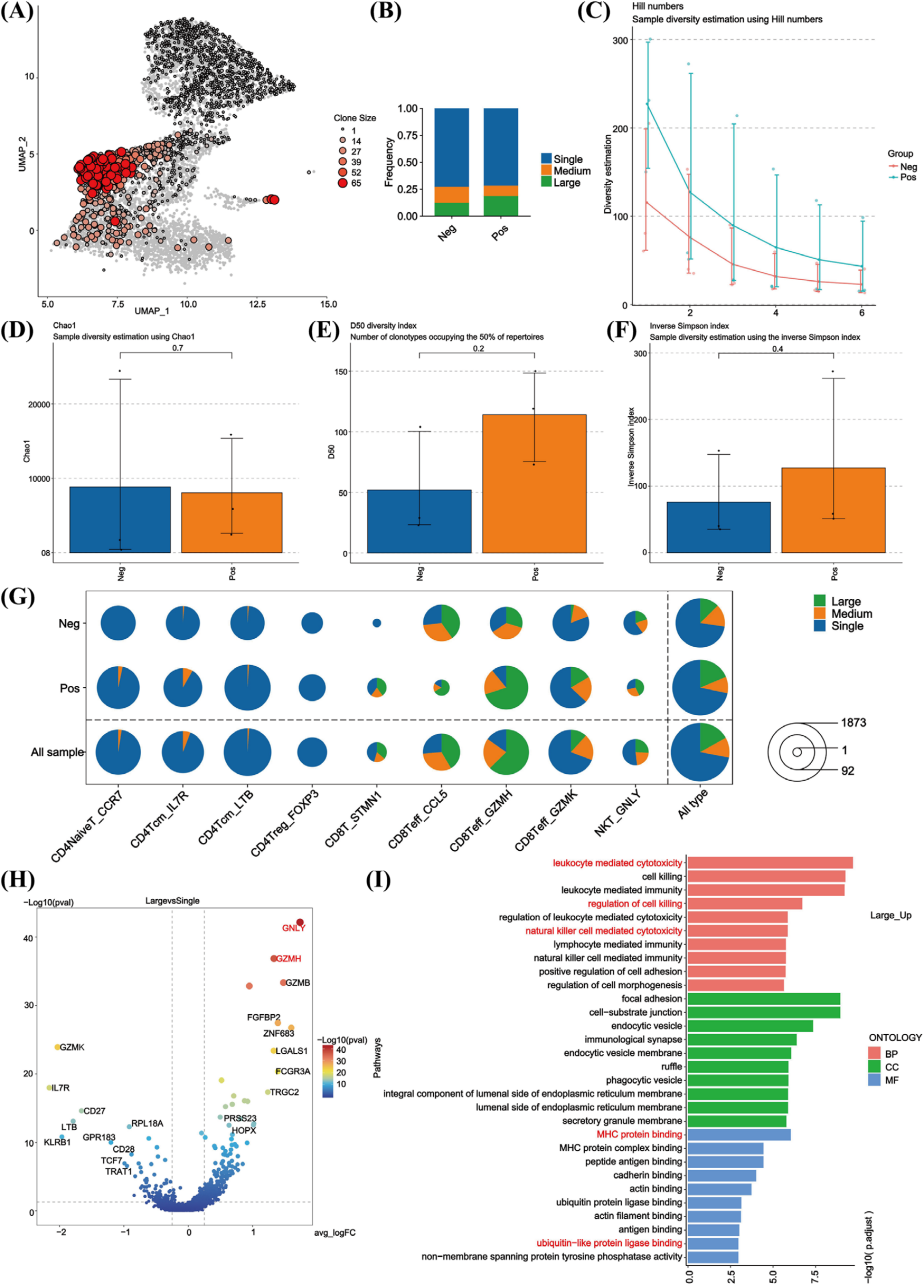
T‐cell clone amplification and TCR library diversity. (A) Distribution of cloned and amplified T cells. The colour and size of the points reflect the clone size in each cluster. (B) Proportional distribution of cloned and amplified T‐cell clusters between groups, classification by clone size (single: clone size = 1; medium: 1 < clone size ≤ 10; large: clone size > 10). (C‒F) Sample diversity estimation using Hill numbers. TCR diversity quantification using Chao1, D50 and Inverse Simpson, and corresponding *p*‐values calculation using two‐sided Wilcoxon test. (G) Percentage of T‐cell clone size in positive (Pos) and negative (Neg) groups. The size of the circle indicating the total number of clones. (H) Volcano plot representing differentially expressed genes (DEGs) between large and single clone size. (I) Gene Ontology (GO) enrichment analysis revealing upregulated pathways of large clone size.

scTCR‐seq described Pos and Neg characteristics of T‐cell receptors and showed the distribution of cloned and amplified T cells (Figure 4A). An elevated proportion of large clonal T cells and diversity of T lymphocytes in peripheral blood estimated by Hill numbers were observed in Pos group, but not by other approaches for evaluating diversity (Figure 4B‒F). The percentage of large clonal T cells was greater in CD8Teff_GZMH and NKT_GNLY cells, especially in Pos group (Figure 4G). Comparing DEGs of large and single clonal T cells revealed that large clonal cells significantly expressed GZMH and GNLY and were enriched in leukocyte‐mediated cytotoxicity, NK‐cell‐mediated cytotoxicity, regulation of cell killing, MHC protein binding and ubiquitin‐like protein ligase binding, coinciding with the characteristics exhibiting high cytotoxicity and exhaustion in Pos group as described previously (Figure 4H,I).

### 3.5 Overview of B cells in peripheral blood

Although B cells were divided into 10 subgroups, three cell clusters including SwitchedMemoryBCells and UnSwitchedMemoryBCells enriched in Pos group and NaiveB enriched in Neg group were identified according to typical markers (Figure S4A‒F). GO enrichment analysis of DEGs upregulated by NaiveB in Neg group showed enrichment in neutrophil chemotaxis, neutrophil migration and leukocyte chemotaxis, suggesting that NaiveB might form an anti‐TME by recruiting natural immune cells such as neutrophils to reduce the risk of metastasis (Figure S4G,H).

### 3.6 LGALS9‒HAVCR2 immune checkpoint interaction existing between peripheral blood immune cells in Pos group

**FIGURE 5:**
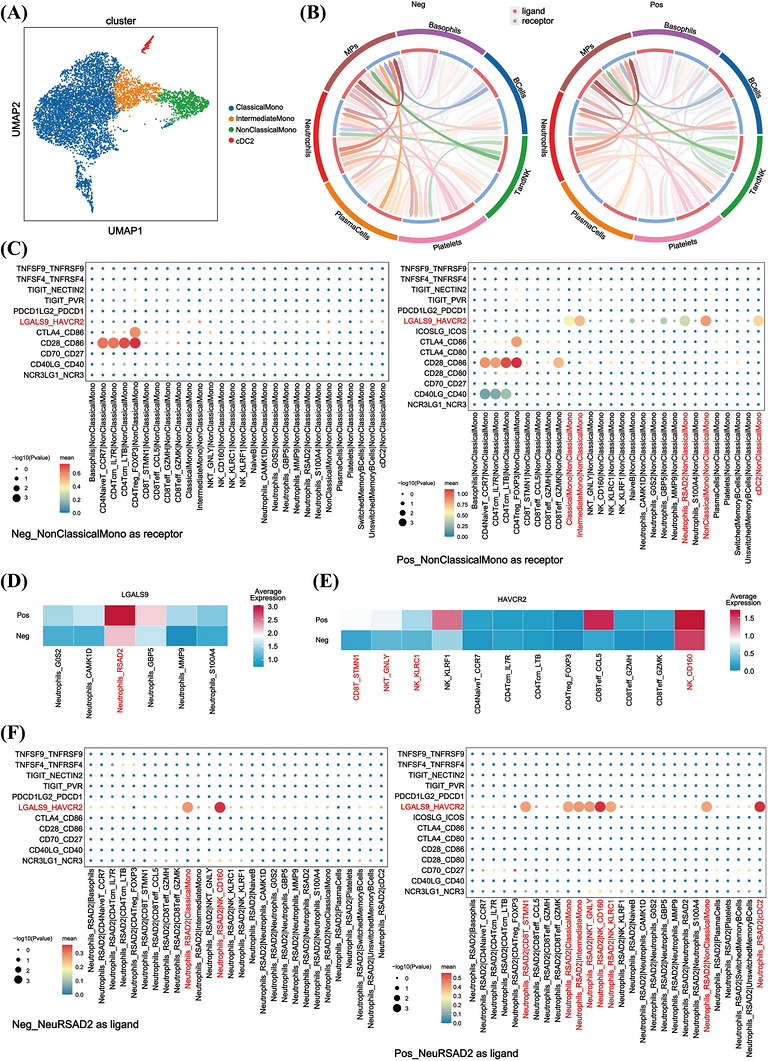
LGALS9‒HAVCR2 enriched in neutrophil‒mononuclear phagocyte (MP)‒TandNK axis. (A) Visualisation of all annotated MP clusters. (B) Chord plot representing the quantity of intercellular interactions of MPs. (C) Dot plot showing the immune checkpoint interactions of non‐classical monocytes (NonClassicalMono) as receptor in negative (Neg) and positive (Pos) groups. (D) Dot plot showing the immune checkpoint interactions of neutrophils_RSAD2 as ligand in Neg and Pos groups. (E) Heatmap showing the expression of LGALS9 gene of neutrophil subgroups in Neg and Pos groups. (F) Heatmap showing the expression of HAVCR2gene of TandNK subgroups in Neg and Pos groups.

**FIGURE 6:**
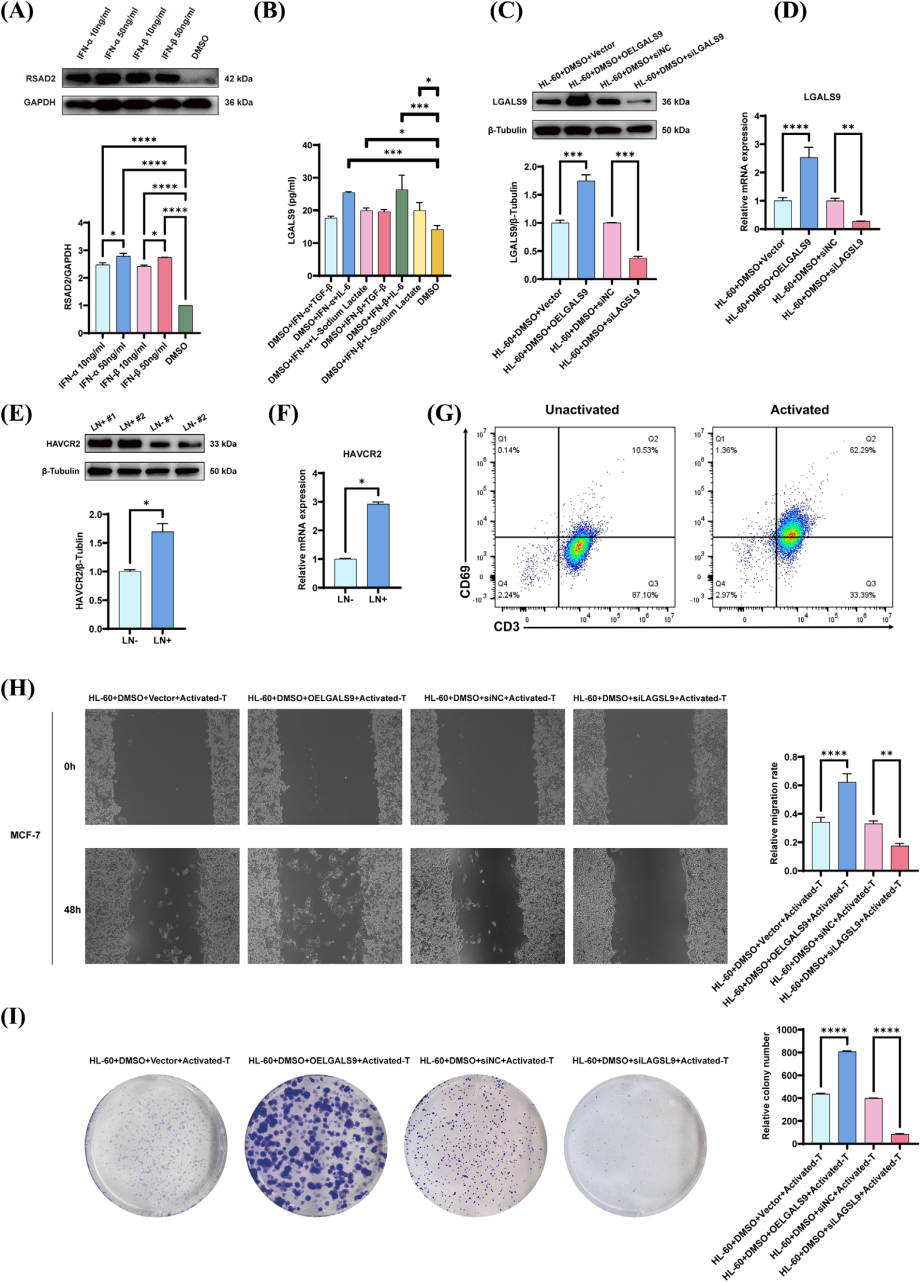
Co‐culture system verified the immune escape effect produced by LGALS9‒HAVCR2 of neutrophil‒T cell axis. (A) Western blot (WB) assay and statistics of RSAD2 and GAPDH of HL‐60 cells induced by DMSO. Neutrophil‐like cells were added with different type I interferon and concentrations as experimental group, and HL‐60 cells after 5 days of 1.25% DMSO as standard group. One‐way analysis of variance (ANOVA) test was conducted. (B) Enzyme‐linked immunosorbent assay (ELISA) assay of LGALS9 secretion in neutrophil‐like cells in different impact factors to simulate peripheral blood environment of breast cancer (BC) patients with lymph node (LN) metastasis. One‐way ANOVA test was conducted. (C) WB analysis and statistics of LGALS9 and β‐Tubulin in HL‐60 cells induced by DMSO. Neutrophil‐like cells were transferred into plasmid to overexpress LGALS9, with Vector as control; siLGALS9 to knockdown LGALS9, with siNC as the control. One‐way ANOVA test was conducted. (D) Quantitative reverse transcription polymerase chain reaction (qRT‐PCR) analysis and statistics of the overexpression and knockdown efficiency of LGALS9 in neutrophil‐like cells, with ACTB as internal reference. One‐way ANOVA test was conducted. (E) WB analysis and statistics of HAVCR2 and β‐Tubulin in peripheral blood of Pos and Neg BC patients. One‐way ANOVA test was conducted. (F) qRT‐PCR analysis and statistics of HAVCR2 in peripheral blood of Pos and Neg BC patients, with ACTB as internal reference. One‐way ANOVA test was conducted. (G) Representative flow cytometry images and statistical of CD3+CD69+ activated T cells. T test was conducted. (H and I) Wound‐healing and colony formation assay and statistics of verifying the immune escape effect of LGALS9‒HAVCR2 on neutrophil‒T cell axis. The neutrophil‐like cells overexpressing or knocking down LGALS9 were co‐cultured with activated T cells and MCF‐7 BC cells. One‐way ANOVA test was conducted.

Given the establishment of an exhausted and immunosuppressive environment in the peripheral blood of metastatic patients, we hypothesised that certain immune checkpoint interactions might be upregulated. Classical monocytes (ClassicalMono), intermediate monocytes (IntermediateMono), non‐classical monocytes (NonClassicalMono) and conventional dendritic cells 2 (cDC2) were identified by transcriptional profiling of peripheral blood MPs, and the proportions of ClassicalMono and IntermediateMono in Pos group increased, while NonClassicalMono and cDC2 in Neg group increased (Figures 5A and S4I‒M). Using CellphoneDB to predict interactions, MPs interacted more with neutrophils, TandNK cells and within MPs in Pos group than in Neg group (Figure 5B). We focused on immune checkpoint interactions and discovered that Pos group had more LGASL9‒HAVCR2 interaction pair of all MPs subgroups whether as ligand or receptor and higher expression of LGALS9 and HAVCR2 genes in subclusters of MPs (Figures 5C and S4N‒R). In addition to MPs, we wondered whether the LGALS9‒HAVCR2 interaction pair would also occur among other immune cells in Pos group. We observed at the expression of LGALS9 and HAVCR2 genes in Pos group and found that they were not only predominantly enriched in neutrophils, TandNK cells and MPs of large class annotations but also neutrophil_RSAD2 with high ISG signature and CD8_STMN1, NKT_GNLY, NKCD160 and NK_KLRC1 with high proliferation, cytotoxicity and exhaustion signatures of subdivision annotations (Figures 5D,E and S4S). Cell‐to‐cell interactions showed that only as ligand, neutrophil_RSAD2 enriched LGALS9‒HAVCR2 with MPs subgroups and the above TandNK cells in Pos group (Figure 5F). Intercellular interaction analysis revealed that only when CD8_STMN1, NKT_GNLY, NK_CD160 and NK_KLRC1 acted as receptor could they express LGALS9‒HAVCR2 interactions with neutrophil_RSAD2 and subclusters of MPs (Figure S4T‒U). We speculated that neutrophils with ISG (NISG) could secrete LGALS9 to bind to other immune cells in the peripheral blood environment of Pos BC patients. To functionally validate whether NISG could serve as the source of LGALS9 in the metastatic context, we performed in vitro experiments using the HL‐60 differentiation model. We demonstrated that neutrophil‐like cells differentiated by HL‐60 cells induced by DMSO highly expressed RSAD2 after IFN‐I stimulation, and more significant expression with higher concentration (Figure 6A). And then we simulated the environment of metastasis‐positive peripheral blood by adding TGF‐β, IL‐6 and L‐sodium lactate, and measured the concentration of LGALS9 by ELISA and found that more LGALS9 could be secreted by NISG in the environment of IL‐6 and L‐lactate (Figure 6B). We then established a model by modulating LGALS9 expression in HL‐60‐derived neutrophil‐like cells via overexpression (OE‐LGALS9) or siRNA‐mediated knockdown (siLGALS9), with respective controls (Vector and siNC) (Figure 6C,D). Then, the results from PBMC first confirmed the amplification and purification effect of T cells, and validated that activated T cells from Pos group indeed exhibited higher baseline protein expression of the exhaustion receptor HAVCR2 compared to those from Neg group (Figure 6E,F). Then, we performed co‐culture experiments to directly test the functional consequence of the LGALS9‒HAVCR2 interaction observed in our single‐cell data. When these engineered neutrophil‐like cells were co‐cultured with activated T cells from LN donors and MCF‐7 BC cells, a clear relationship emerged. Flow cytometry first verified that T cells have been activated (Figure 6G), And the activated T cells exposed to OE‐LGALS9 neutrophils lost their capacity to suppress tumour growth, as evidenced by increased MCF‐7 viability, enhanced clonogenic survival and accelerated migration. Correspondingly, neutralisation of LGALS9 via knockdown in neutrophils restored the tumouricidal function of co‐cultured T cells, leading to inhibition of MCF‐7 proliferation and migration (Figure 6H,I). The above results might indicate that in Pos group NISG functioned as the starting point of LGALS9‒HAVCR2 to secrete LGALS9 binding to HAVCR2 on the surface of MPs and TandNK cells, which transformed the cells that were originally lethal to the tumour cells into an exhausted state without effector ability, thus forming an immunosuppressive environment that allowed the tumour cells to escape and metastasis.

### 3.7 Tumour tissue results validating the observed immune response in peripheral blood

**FIGURE 7:**
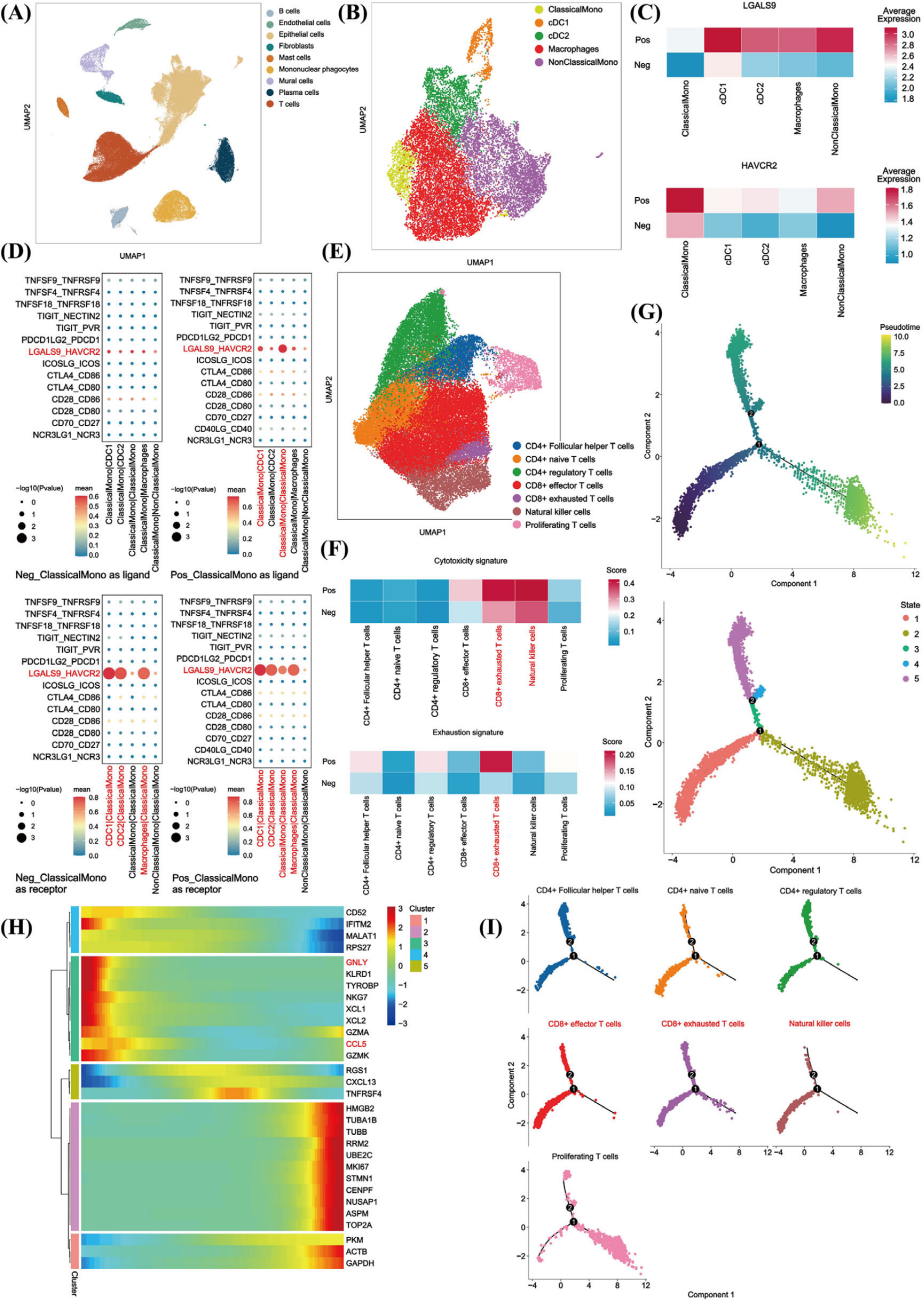
Validation of immune responses through breast cancer tissues. (A) Visualisation of all annotated cell clusters. (B) Visualisation of all annotated mononuclear phagocyte (MP) clusters. (C) Heatmap showing the expression of LGALS9 and HAVCR2 genes of MPs in positive (Pos) and negative (Neg) groups. (D) Dot plot showing the immune checkpoint interactions of ClassicalMono in breast cancer (BC) tissues as ligand and receptor in Neg and Pos groups. (E) Visualisation of all annotated T‐cell clusters. (F) Heatmap showing cytotoxicity and exhaustion signatures characterising T‐cell clusters. (G) Pseudo‐time trajectory of T cells showing five different states and two fate‐determining directions. (H) The expression of the genes in a state‐dependent manner. Each row indicating the standardised kinetic curves of a gene. (I) Pseudo‐time trajectory analysis of T‐cell clusters.

To determine whether the pro‐metastatic immune landscape observed in peripheral blood was also reflected within the local tumour ecosystem, we analysed single‐cell transcriptomes of validation cohort of primary BC tissues from 14 patients. A total of 10 cell types, B cells, endothelial cells, epithelial cells, fibroblasts, mast cells, MPs, mural cells, plasma cells, plasmacytoid dendritic cells and TandNK cells, were annotated with reference to the typical labelling in the mammary gland (Figures 7A and S5A). To further validate previous literature and our in vitro simulation rationale, we analysed the expression of inflammatory factors and hypoxia‐related markers in malignant epithelial cells (Figure S5B). Malignant epithelial clusters identified by inferCNV analysis from Pos group showed significantly elevated expression of inflammatory cytokines including IL‐6 and TGFB1 and hypoxia‐associated genes such as LDHA and HIF1A compared to Neg group (Figure S5C‒E). In addition, we extracted RNA‐seq data of luminal B subtype BC patients in TCGA‒BRCA, and also observed that the expression of above factors in Pos group was significantly higher than Neg group (Figure S5F). LGALS9‒HAVCR2 interaction pair occurred more between MPs and other cells in Pos group, and we subsequently made further subdivision annotations of MPs, including ClassicalMono, cDC1, cDC2, macrophages and NonClassicalMono, and similar to the results in peripheral blood, not only were the LGALS9‒HAVCR2 interactions more enriched in Pos group, but also LGALS9 and HAVCR2 genes were more expressed in Pos group (Figures 7B‒D and S5G). Observing the expression of specific gene sets in 10 subclusters of TandNK cells, we identified seven cell types, including CD4^+^ follicular helper T cells, CD4^+^ naive T cells, CD4^+^ regulatory T cells (Tregs), CD8^+^ effector T cells, CD8^+^ exhausted T cells, NK cells and proliferating T cells (Figures 7E and S5H,I). UCell scoring showed that in Pos group, CD8^+^ effector T cells, CD8^+^ exhausted T cells and NK cells were more enriched with cytotoxicity and exhaustion signatures and CD4^+^ Tregs were more enriched with regulatory signature (Figures 7F and S5J). Compared to Neg group in Pos group, FOXP3 gene was more expressed in CD4^+^ Tregs, CCL5 in CD8^+^ effector T cells, GZMH in CD8^+^ exhausted T cells, and GNLY in NK cells, all of which were consistent with previous findings (Figure S5K). Pseudo‐time analysis still classified TandNK cells into five states and two fate‐determining directions, and like peripheral blood, CCL5 and GNLY were also highly variable genes and CD8^+^ effector and exhausted TandNK cells were also enriched in early differentiation (Figures 7G‒I and S5L,M).

**FIGURE 8:**
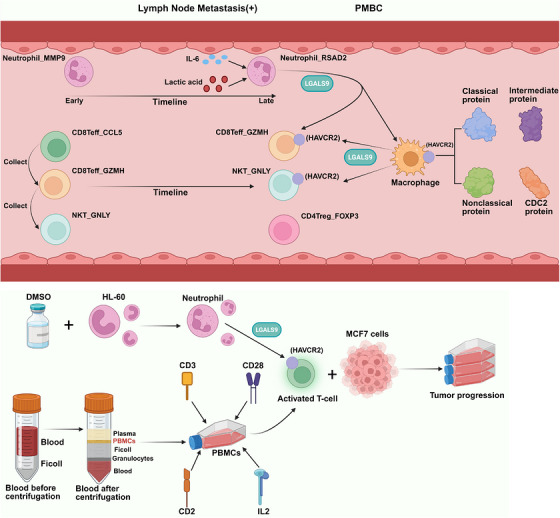
Summary of the role of LGALS9‒HAVCR2 in peripheral blood of breast cancer (BC) patients with lymph node metastasis.

And as schematically summarised in Figure 8, our findings delineated a novel workflow to provide a comprehensive framework that set the stage for elucidating the underlying mechanisms and functional implications in the following discussion.

## 4 DISCUSSION

Variations in peripheral blood immune cells of Pos and Neg BC patients were compared at the single‐cell level by high‐throughput sequencing, and the characteristic changes of PBMC were confirmed using immune cells from BC tissues. The results of scRNA‐seq of peripheral blood showed that in Pos group, although the proportion of neutrophils decreased, NISG among them increased and secreted LGALS9 to act on HAVCR2 on the surface of MPs and TandNK cells, which resulted in cytotoxic TandNK cells exhibiting exhaustion; in addition, multiple TandNK cells with specific features were enriched in Pos and Neg groups and the observed immune changes were verified by scTCR‐seq, in vitro experiments and scRNA‐seq of tissues. Changes in neutrophils, TandNK cells, B cells and MPs in peripheral blood in Pos group reflected the remodelling of the systemic immune response and the establishment of immunosuppressive microenvironment. LN metastasis implies increased tumour burden and sustained release of tumour antigens into the circulation, which will continuously stimulate the adaptive immune system such as T and B cells and the innate immune system such as NK cells and MPs.^36^, ^37^ The relative decrease of neutrophil proportion might be due to the dynamic balance change caused by functional polarisation, which manifested in enrichment of specific pro‐metastatic subclusters in Pos group.

Neutrophils proliferate and mature in the bone marrow and are released into the peripheral blood circulation to become the most abundant leukocyte type as well as the major immune cells,^38^, ^39^ and are recruited to form TANs induced by various tumour‐derived chemokines in TME.^40^ Based on phenotypic differences in previous studies, TANs could be divided into immature neutrophils (NI), anti‐tumourigenic (N1 type), pro‐tumourigenic (N2 type) and NISG.^39^, ^41^ Neutrophil_RSAD2 annotated in PBMC of this study highly expressed ISG and was enriched in response to IFN‐I and IFN‐I pathway, raising the question of whether the normal neutrophils in blood circulation were transformed into NISG after IFN‐I stimulation. Due to the short lifespan of neutrophils,^42^ previous study has proved that DMSO‐induced HL‐60 cells can be used as research model for neutrophils.^43^ However, we acknowledge that our in vitro experiments employed DMSO‐differentiated HL‐60 cells as a surrogate for human neutrophils. Although this model is widely used due to the short lifespan and low yield of primary neutrophils, it cannot fully recapitulate the phenotypic complexity, precise secretory profiles, or plasticity of genuine patient‐derived TANs or NISGs. IFN‐I binds to the neutrophil membrane receptor IFNAR1/IFNAR2 and activates the receptor‐associated kinase JAK,^44^ which phosphorylates STAT1 and STAT2, both of which bind to the transcription factor IRF9 to create the ISGF3 complex, which moves into the nucleus and attaches to the interferon‐stimulated response element in the ISG promoter to boost ISG production.^45^ One of the characteristics of TANs in TME is that their functions are not fixed but can be interconverted between anti‐tumourigenic and pro‐tumourigenic forms through polarisation process,^46^ for example, cytokines such as TGF‐β and IL‐6 can induce the polarisation of TANs to N2 type^47^; another major feature is that the hypoxic environment formed by TME promotes the secretion of PD‐L1 by neutrophils through the production of lactic acid by glycolytic pathway,^48^, ^49^ and the combination of PD‐L1 and typical exhausted PD‐1 on T cells forms an immunosuppressive environment to promote immune escape and metastasis.^50^ We found that only neutrophil_RSAD2 as ligand had LGALS9‒HAVCR2 interactions with MPs and TandNK cells; therefore, we speculated that NISG in peripheral blood environment after LN metastasis could secrete LGALS9. TGF‐β, IL‐6 and L‐sodium lactate were added to simulate the environment after LN metastasis and it was observed that more LGALS9 secreted by NISG was detected only in the environment with increased IL‐6 and lactate. Previous studies have shown that IFN‐I and IL‐6 inhibited anti‐tumour immunity by inducing IRF1 and thus upregulating PD‐L1,^51^, ^52^ lactate promoted LGALS9‐mediated immunosuppression by inhibiting the nuclear factor‐kappa B signalling pathway,^53^ and TGF‐β enhanced SHP1 phosphatase activity in a way depended on AKT‒SMAD3, decreased the tyrosine phosphorylation of STAT1, and inhibited STAT1 dependent immune evasion‐associated molecules such as PD‐L1 and LGALS9 expression,^54^ thus indicating that the secretion of LGALS9 by NISG is influenced by the complicated immunoregulatory network. Our analysis of tumour tissues further revealed that malignant epithelial cells in Pos group are a significant source of inflammatory and hypoxic signals, thereby offering a direct explanation for the specific factors driving the immunosuppressive LGALS9‒HAVCR2 axis in the periphery. This confirms that the primary TME in metastatic disease is intrinsically more inflammatory and hypoxic, providing a direct source for the soluble factors that likely contribute to the systemic immune alterations observed in the peripheral blood. On the one hand, MMP9 secreted by neutrophils in Pos group acts as a key protease to degrade and remodel ECM to support tumour cell colonisation and form metastatic focus^55^; on the other hand, it directly releases vascular endothelial growth factor to promote the formation of neovascularisation and thus increases tumour cell‒lymphatic system contact to promote tumour growth.^56^ Combined with pseudo‐time trajectory analysis further revealed that the rapid response of neutrophil_MMP9 in the early phase and the sustained impact of neutrophil_RSAD2 in the late phase together formed a continuous spatiotemporal collaborative pattern to maintain immunosuppression to promote metastasis.

Tregs are suppressive CD4^+^T cells with FOXP3 as the symbolic transcription factor,^57^ which can promote the escape of tumour cells by inhibiting antigen‐presenting cells (APCs) from presenting antigens to effector T cells, releasing regulatory cytokines into microenvironment and inducing the expansion of existing Treg cells to form an immunosuppressive environment.^58^ Previous studies have found that the strong inhibitory ability and remarkable contribution to tumour development of Treg cells were mainly due to their influence on the environment,^16^, ^59^ and our research found that Treg cells expressed high levels of FOXP3 and exerted immunosuppressive functions in Pos group in both peripheral blood and tumour tissue environments, which shows that Treg cells may not only serve as a prognosticator of poor prognosis, but also as a potential target for future therapies.^60^ GZMH could produce tumour cell killing by damaging DNA and mitochondria as well as generating reactive oxygen species,^61^ and GNLY was thought to co‐localise to cytotoxic TandNK cells and exert toxic effects against tumour cells.^62^ Previous study has shown that exhausted T cells might result from continuous exposure to tumour antigens after failure of tumour cell clearance^63^; in our study, large clonal T cells were enriched in GZMH/GNLY, which demonstrated that TCR signal persisted, and the expression of inhibitory checkpoint receptors such as HAVCR2 in CD8Teff_GZMH and NKT_GNLY in peripheral blood increased, indicating that TandNK cells with dual cytotoxic‐exhausted characteristics lost the ability to kill tumours thus promoting immune escape,^64^ and CD8^+^effector, exhausted TandNK cells enriched for toxicity and exhaustion characteristics in tumour tissues affirmed that the dual effect might serve as a prognostic feature. The role of CCL5 in BC is controversial, previous report has shown that CCL5 was associated with BC metastasis,^65^ but a recent research showed that HSF1 could inhibit anti‐tumour immunoreactivity in BC by inhibiting CCL5 to reduce the recruitment of CD8^+^T cells.^66^ Our results were similar and revealed that CCL5 played a role in the recruitment of CD8^+^T cells and NK cells to the peripheral blood and tumour sites to form immune surveillance as a chemokine. Flow cytometry demonstrated that CD8Teff_CCL5 was enriched in Neg group, whereas NKT_GNLY in Pos, suggesting that differential factors may serve as potential predictors on LN metastasis; in addition, chemokine effector T cells and cytotoxic‐exhausted T cells were located in the early stage of differentiation in both peripheral blood and tumour tissues, which further showed that the anti‐tumour environment was formed under the joint action of immune monitoring function deficiency and exhaustion leading to reduced effector capacity. Our pseudo‐time trajectory analysis revealed a dynamic differentiation continuum among immune cells. In TandNK cells, CCL5+ anti‐metastatic and GNLY+ cytotoxic‐exhausted populations were enriched early, indicating that functional fate is determined early in differentiation. The LGALS9‒HAVCR2 axis, originating from late‐stage neutrophil_RSAD2 as key LGALS9 secretor, then reinforces the exhaustion of GNLY+ T/NK cells, creating a feed‐forward loop that sustains immunosuppression. Thus, the differentiation pathways not only reflect cellular maturation but also encode functional specialisation that coordinates the systemic immune landscape in LN metastasis. The double‐sided role of annotated B lymphocytes reflected the environmental differences in peripheral blood in Neg group. The functions of NaiveB cells include recruiting immune cells such as neutrophils in association with the anti‐tumour environment; SwitchedMemoryB cells and UnswitchedMemoryB cells formed after encountering tumour antigens have long‐term survival ability and provide faster and more effective immune response.^67^

Galectin‐9 (Gal‐9), which LGALS9 encodes, regulates the immune response by inhibiting cytotoxicity of T lymphocytes and NK cells. Gal‐9 in tumour evades immune surveillance by acting on Tim‐3, PD‐1 receptors on T cells or NK cells to induce programmed death or reduce cytotoxicity.^68^, ^69^ Previous study has shown that highly expressed Gal‐9 tumour‐associated macrophages were associated with poorer prognosis and exhausted CD8^+^T cells labelled with PD‐1 and TIM‐3.^70^ Tim‐3 (HAVCR2), similar to PD‐1, is the most exhausted type of CD8^+^T cells in tumour infiltration, and its expression on NK cells has been associated with functional exhaustion and tumour progression.^71^ The interaction of Gal‐9 and Tim‐3 induces the binding of released Bat3 to Fyn, which encourages C‐terminal c‐Src kinase (Csk) recruitment by phosphorylating the phosphoprotein associated with glycosphingolipid microdomains 1 (PAG), which suppresses T‐cell function by adversely regulating Lck activity.^72^ Both NISG and MPs could be seen to secrete Gal‐9 as ligand through cell‐to‐cell interactions, and multiple pathways have been suggested to explain the mechanism of Gal‐9 leaving cells. Galectin is synthesised in the cytoplasm, independent of the conventional endoplasmic reticulum/Golgi transport mechanism, and is secreted through a nonclassical pathway.^73^, ^74^ Interestingly, in addition to having Tim‐3 on T and NK cells with dual cytotoxic‐exhausted characteristics as receptor to bind the predominant ligand Gal‐9, there was also in various subclusters of MPs to occur LGALS9‒HAVCR2 interactions in Pos group. Previous study has shown that Tim‐3 upregulation in MPs correlated with tumour progression,^75^ and we speculate that Gal‐9/Tim‐3 make macrophages undergo M2‐type polarisation by secreting inhibitory cytokines in order to enhance immunosuppression.^76^, ^77^, ^78^ Our study suggests that the LGALS9‒HAVCR2 axis may represent a critical immunosuppressive hub associated with BC metastasis. Our in vitro co‐culture experiments suggest that neutrophil‐derived LGALS9 can impair T‐cell cytotoxicity. The reversal of this phenotype upon LGALS9 knockdown provided a proof‐of‐concept that targeted disruption of this axis could rescue T‐cell function. This mechanistic may support the development of therapeutic strategies aimed at this checkpoint, potentially to reverse immune exhaustion and enhance the efficacy of existing immunotherapies in metastatic BC. The landscape of immune response established by previous single‐cell sequencing studies similarly showed that biomarkers of immune cells could be used as prognostic markers,^79^, ^80^ as well as elevated expression of LGALS9‒HAVCR2 interactions were found to be prevalent in poor prognosis.^19^ In summary, we conclude that LGALS9‒HAVCR2 in peripheral blood can be delivered in the neutrophil‒MP‒TandNK axis, and Gal‐9 and Tim‐3 enriched in Pos group can used as immunosuppressive markers to predict the prognosis of LN metastasis.^81^

Our study identifies specific peripheral blood immune subsets and checkpoint interactions associated with LN metastasis. The translational advantage of these findings lies in their basis in peripheral blood, a readily accessible biofluid.^19^, ^82^ This positions our identified signatures—such as the abundance of interferon‐stimulated neutrophil_RSAD2 and exhausted NKT_GNLY+ cells, and the systemic activity of the LGALS9‒HAVCR2 axis—as promising candidates for the development of minimally invasive liquid biopsy tools.^83^ To realise this potential, the defined path involves transforming single‐cell observations into a clinically applicable assay. This process entails developing a robust, reproducible assay to quantify the key cellular or molecular features in blood and prospectively validating the assay's performance for its intended use, such as predicting metastatic risk prior to surgery or monitoring for early signs of recurrence.^84^ Future studies focused on these translational steps are warranted to evaluate whether this systemic immune profiling can augment current clinical staging and ultimately improve personalised management for BC patients. While further validation in large, prospective cohorts is needed, our work provides a compelling immunological foundation for such blood‐based biomarker development.

Our study focused exclusively on the luminal B subtype. While the immune landscape varies across BC subtypes, with basal‐like tumours exhibiting the richest immune infiltration and luminal tumours showing relatively lower immune presence,^85^ the core mechanisms identified here—LGALS9‒HAVCR2‐mediated T‐cell exhaustion and neutrophil immunosuppressive polarisation—may have broader relevance. T‐cell exhaustion signatures are differentially expressed across multiple cancer types and correlate with patient survival,^86^ and LGALS9 is widely expressed across various malignancies, where it regulates immune homeostasis and tumour pathogenesis through HAVCR2 interaction.^73^ Moreover, immunosuppressive neutrophil subpopulations have been identified across 21 cancer types in pan‐cancer single‐cell analyses, indicating that neutrophil‐driven immune suppression is a conserved phenomenon beyond BC.^87^ Future studies across different BC subtypes and other cancer types are warranted to validate the generalisability of our findings. Prospective studies incorporating multiple BC subtypes and other malignancies are needed to determine whether the identified immune signatures represent subtype‐specific phenomena or broadly conserved mechanisms of LN metastasis‐associated immune remodelling.

Undeniably, there were still some limitations in our research. First, the small sample size remained a key limitation affecting statistical validity, generalisability of conclusions. In the future, larger and independent multicentre cohorts are needed to validate the core findings and assess the prognostic value. Second, the study clarified the source and receptor cells of LGALS9‒HAVCR2, but has not yet resolved the specific molecular mechanisms of how the neutrophil‒MP‒TandNK axis regulated exhaustion and dysfunction in the metastatic environment. While our in vitro co‐culture experiments established a causal role for neutrophil‐derived LGALS9 in promoting T‐cell exhaustion via HAVCR2, in vivo validation using T‐cell‐specific genetic manipulation remains unaddressed in the current study. Future studies employing conditional knockout mice or performing adoptive transfer of engineered T cells will be essential to further validate this immunosuppressive axis and explore its therapeutic potential. Third, the study provided the basis for peripheral blood‐based diagnostics, but has not yet been translated into a specific and clinically applicable detection index. In future prospective cohorts, blood‐based multimodal diagnostic/prognostic models need to be developed and validated for easier prognostic determination. Fourth, another limitation of this study is the lack of comparison with healthy donors, which prevents us from distinguishing immune alterations specific to metastasis from those general to BC. Future studies incorporating healthy controls will be valuable to provide a complete spectrum of immune changes. While our study identifies a distinct peripheral immune landscape associated with LN metastasis, we acknowledge the potential influence of confounding variables and unmeasured demographic or lifestyle factors cannot be entirely ruled out. Our cross‐sectional design cannot completely explain whether the immune profile is a cause or a consequence of metastasis. Future prospective studies, ideally with serial blood sampling from diagnosis through metastasis, and with comprehensive molecular profiling of paired tumours, are needed to establish causal relationships and control for these potential confounders. Last, we acknowledge that profiling PBMCs cannot fully recapitulate the spatial and functional complexity of immune interactions within the tumour‐draining LNs or the primary TME. Future studies that provide the mutational and genomic context integrating primary tumours, matched LN metastases and PBMCs would be invaluable to build a more comprehensive, systems‐level understanding of the mechanisms driving immune remodelling and metastasis.

## 5 CONCLUSION

In this study, we analysed the differences of immune cells in PBMC and BC tissues with and without LN metastasis by scRNA‐seq, scTCR‐seq and in vitro experiments. We identified pro‐metastatic and anti‐metastatic subpopulations and tracked the key targets that formed the immunosuppressive environment. Our study contributes to a more comprehensive understanding of the systemic immune remodelling in BC patients with LN metastasis and identifies candidate immune signatures that may serve as biomarkers after prospective validation in larger cohorts.

## AUTHOR CONTRIBUTIONS

Bo Chen: Conceptualization (lead); supervision (lead); validation (lead); writing–review and editing (lead); Kang Ma: Conceptualization (equal); data curation and analysis(equal); writing–original draft (lead); Yunjie Wang: Conceptualization (equal); data curation and analysis(equal); writing–original draft (lead); Liulu Zhang: Investigation (equal); methodology (equal); project administration (equal); Xinyue Feng: Investigation (equal); methodology (equal); project administration (equal); Cheng Long: Investigation (equal); methodology (equal); project administration (equal); Xuejing Tan: Investigation (equal); methodology (equal); project administration (equal); Kun Wang: Conceptualization (lead); supervision (lead); validation (lead); writing–review and editing (lead).

## CONFLICT OF INTEREST STATEMENT

The authors declare they have no conflicts of interest.

## ETHICS STATEMENT

All study procedures were performed in compliance with the Declaration of Helsinki and approved by the Ethics Committee of Guangdong Provincial People's Hospital (nos. GDREC2014122H and GDREC2019497H). Written informed consent was obtained from each patient before sample collection.

## Supporting information



Supplementary Information

Supplementary Information

Supplementary Information

Supplementary Information

Supplementary Information

Supplementary Information

## Data Availability

The data that support the findings of this study are available from the corresponding author upon reasonable request.
