# Supplementary material for: Single‐cell profiling reveals peripheral blood immune landscape remodelling in breast cancer lymph node metastasis
Source: Clin Transl Med. 2026 Jun 9;16(6):e70686. doi: 10.1002/ctm2.70686 (PMC13247556; doi:10.1002/ctm2.70686)
Supplement: Supplementary file 1 — Supplementary Information [file CTM2-16-e70686-s003.docx]

**Table S1.** **Clinical and pathological characteristics of breast cancer patients in the discovery and validation cohorts.**

| Characteristics | Discovery cohort (PBMC, n=6) | | *P* value | Validation cohort (tumor tissue, n=14) | | *P* value |
| --- | --- | --- | --- | --- | --- | --- |
|  | Neg, n=3 | Pos, n=3 |  | Neg, n=7 | Pos, n=7 |  |
| Age, years (Mean ± SD) | 52.67±8.963 | 50.33±8.386 | 0.4000 | 54.29±7.910 | 48.86±7.603 | 0.5122 |
| T stage, n (%) |  |  | 0.2733 |  |  | 0.5148 |
| T1 | 3 (100) | 2 (66.667) |  |  |  |  |
| T2 |  | 1 (33.333) |  | 6 (85.714) | 5 (71.429) |  |
| T3 |  |  |  | 1 (14.286) | 2 (28.571) |  |
| N stage, n (%) |  |  | **<0.0001*** |  |  | **<0.0001*** |
| N0 | 3 (100) |  |  | 7 (100) |  |  |
| N1 |  | 3 (100) |  |  | 5 (71.423) |  |
| N2-3 |  |  |  |  | 2 (28.577) |  |
| M stage, n (%) |  |  | >0.99 |  |  | >0.99 |
| M0 | 3 (100) | 3 (100) |  | 7 (100) | 7 (100) |  |
| Tumor Grade, n (%) |  |  | >0.99 |  |  | 0.2801 |
| I |  |  |  |  |  |  |
| II | 3 (100) | 3 (100) |  | 4 (57.143) | 2 (28.571) |  |
| III |  |  |  | 3 (42.857) | 5 (71.429) |  |
| ER status, n (%) |  |  | >0.99 |  |  | >0.99 |
| Positive | 3 (100) | 3 (100) |  | 7 (100) | 7 (100) |  |
| PR status, n (%) |  |  | >0.99 |  |  | 0.5148 |
| Positive | 2 (66.667) | 2 (66.667) |  | 5 (71.429) | 6 (85.714) |  |
| Negative | 1 (33.333) | 1 (33.333) |  | 2 (28.571) | 1 (14.286) |  |
| HER2 status, n (%) |  |  | >0.99 |  |  | >0.99 |
| Positive | 2 (66.667) | 2 (66.667) |  | 2 (28.571) | 2 (28.571) |  |
| Negative | 1 (33.333) | 1 (33.333) |  | 5 (71.429) | 5 (71.429) |  |
| Ki-67 index (%, Mean ± SD) | 0.42±0.17 | 0.35±0.16 | 0.4336 | 0.57±0.25 | 0.50±0.27 | 0.8000 |
| Molecular Subtype |  |  | >0.99 |  |  | >0.99 |
| Luminal B | 3 (100) | 3 (100) |  | 7 (100) | 7 (100) |  |
| Naive to any therapy, n (%) | 3 (100) | 3 (100) | >0.99 | 7 (100%) | 7 (100%) | >0.99 |

***There is statistical significance.** Abbreviations: SD: standard error; Neg, lymph node metastasis-negative; Pos, lymph node metastasis-positive; PBMC, Peripheral blood mononuclear cell; ER, Estrogen receptor; PR, Progesterone receptor; HER2, Human Epidermal Growth Factor Receptor 2.
