# Supplementary figures and images for: Single‐cell profiling reveals peripheral blood immune landscape remodelling in breast cancer lymph node metastasis
Source: Clin Transl Med. 2026 Jun 9;16(6):e70686. doi: 10.1002/ctm2.70686 (PMC13247556; doi:10.1002/ctm2.70686)

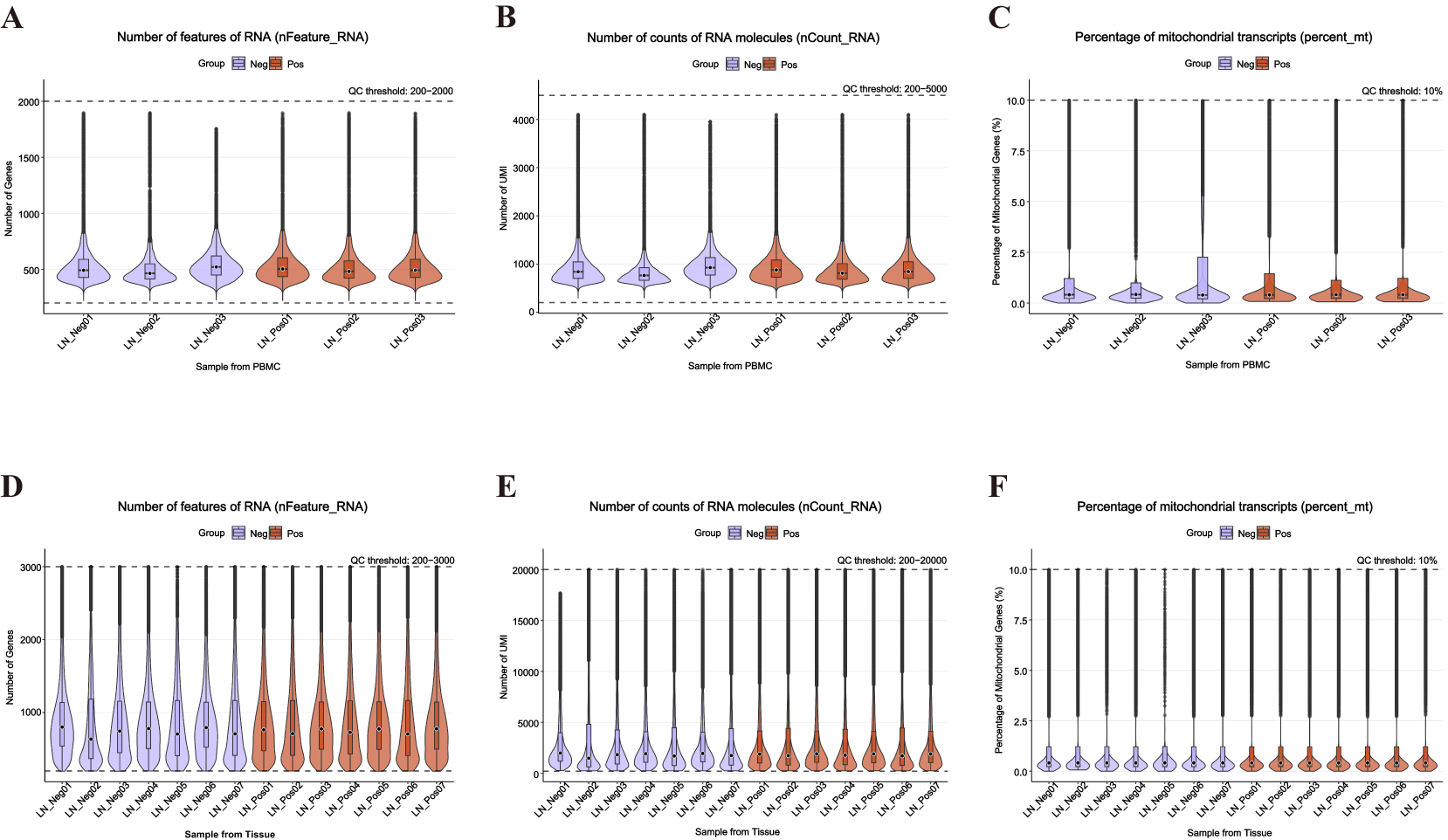

Supplement: Supplementary file 2 — Supplementary Information [file CTM2-16-e70686-s002.tif]

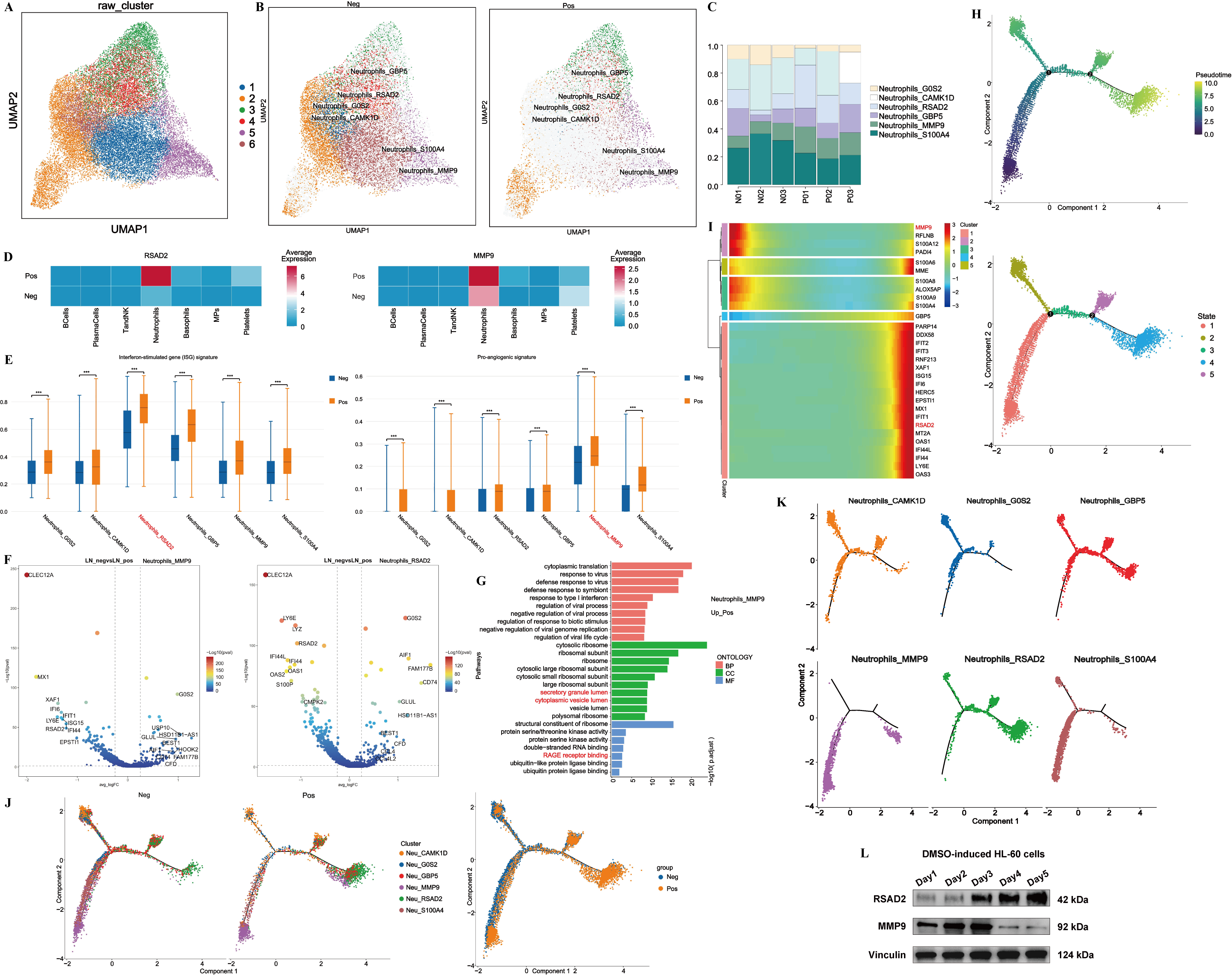

Supplement: Supplementary file 3 — Supplementary Information [file CTM2-16-e70686-s004.tif]

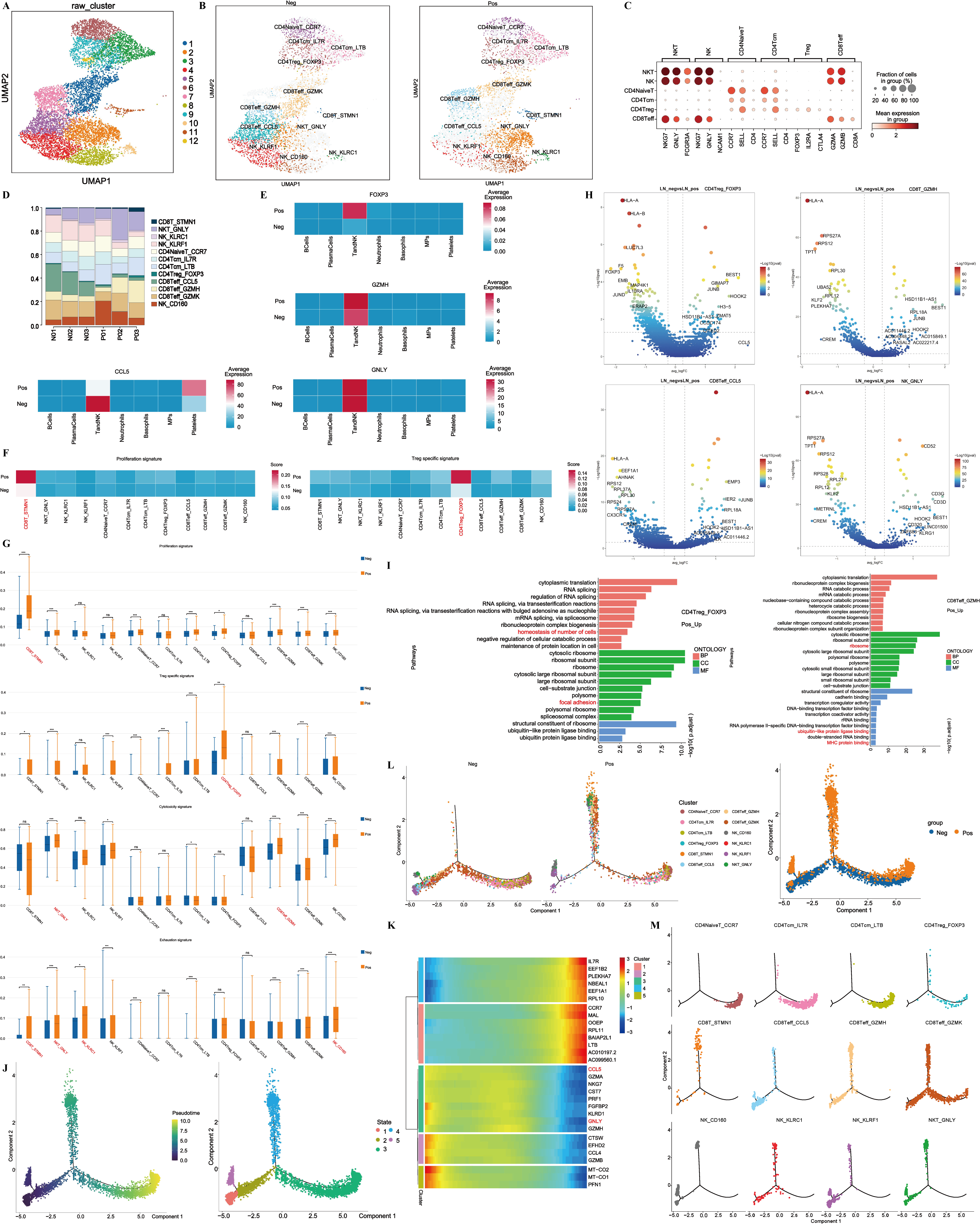

Supplement: Supplementary file 4 — Supplementary Information [file CTM2-16-e70686-s006.tif]

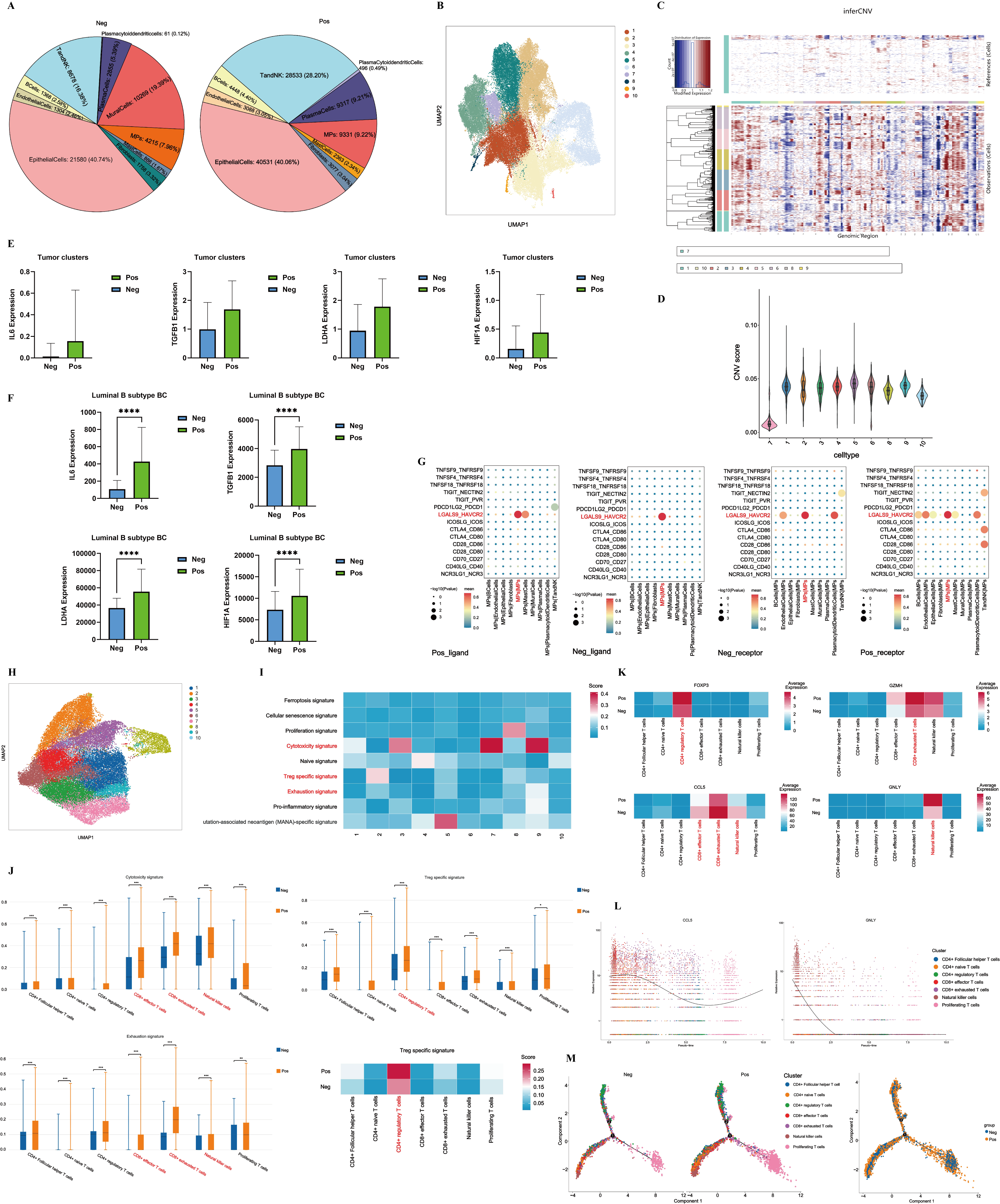

Supplement: Supplementary file 6 — Supplementary Information [file CTM2-16-e70686-s005.tif]
